# Hydrophobins in *Bipolaris maydis* do not contribute to colony hydrophobicity, but their heterologous expressions alter colony hydrophobicity in *Aspergillus nidulans*

**DOI:** 10.3389/ffunb.2025.1604903

**Published:** 2025-11-20

**Authors:** Kenya Tsuji, Hiroshi Yoshida, Masafumi Saba, Yuki Terauchi, Moriyuki Kawauchi, Yoichi Honda, Chihiro Tanaka, Akira Yoshimi

**Affiliations:** 1 Laboratory of Environmental Interface Technology of Filamentous Fungi, Graduate School of Agriculture, Kyoto University, Kyoto, Japan; 2 Laboratory of Terrestrial Microbial Ecology, Graduate School of Agriculture, Kyoto University, Kyoto, Japan; 3 Terrestrial Microbiology and Systematics, Graduate School of Global Environmental Studies, Kyoto University, Kyoto, Japan; 4 Graduate School of Sciences and Technology for Innovation, Yamaguchi University, Yamaguchi, Japan; 5 Laboratory of Forest Biochemistry, Graduate School of Agriculture, Kyoto University, Kyoto, Japan

**Keywords:** hydrophobin, hydrophobicity, *Bipolaris maydis*, *Cochliobolus heterostrophus*, *Aspergillus nidulans*

## Abstract

Hydrophobins are small amphiphilic proteins secreted by filamentous fungi. These proteins confer hydrophobic properties to the hyphae and conidia. *Bipolaris maydis* is the causal agent of southern corn leaf blight; the biological function of its hydrophobins is not clear. In the present study, we focused on the broad function of hydrophobins in the life cycle of this fungus. We found that the *B. maydis* genome encodes four hydrophobins—Hyp1 of class I, and Hyp2, Hyp3 and Hyp4 of class II—and all of them are expressed. We generated single disruptants of each gene, as well as triple and quadruple disruptants. No differences were detected between the wild type and any of disruptants in mycelial growth, conidiation, stress tolerance, virulence, or sexual reproduction. The colony hydrophobicity of all disruptant strains was similar to that of the wild-type strain. Complementation of a null *Aspergillus nidulans* mutant of *dewA*, which showed a significantly reduced colony hydrophobicity, with each of the four *B. maydis* hydrophobin genes restored the hydrophobic phenotype, although the degree of hydrophobicity varied among them. Despite the absence of any significant phenotypic changes in the *B. maydis* mutants generated, results strongly suggest that all four hydrophobins have retained their function in hydrophobicity. Furthermore, the results of this study suggest that the role of hydrophobins might change depending on the fungal species.

## 1 Introduction

Hydrophobins are low-molecular-weight, amphipathic proteins secreted by filamentous fungi (Wösten, 2001). Their amino acid sequences have low similarity, but their hydrophobic patterns are similar. These proteins have a highly conserved pattern of eight cysteine residues (-C-CC-C-C-CC-C-) capable of forming four disulfide bonds (Sehuren and Wessels, 1990; Wösten, 2001). A characteristic feature of hydrophobins is their ability to self-assemble at air–water interfaces (Wösten, 2001). On the basis of their hydrophobic patterns and the solubility of their aggregates in solvents, hydrophobins are primarily classified into classes I and II (Wösten, 2001). Class I hydrophobins are insoluble in sodium dodecyl sulphate (SDS) even at high temperatures but soluble in trifluoroacetic acid, whereas class II hydrophobins are soluble in SDS (Wessels et al., 1991).

Hydrophobins are secreted to the surface of the cell wall of aerial hyphae and conidia and play crucial roles in fungal biological processes (Wösten, 2001). Hyphae of many filamentous fungi are hydrophobic: they repel water and create a hydrophobic barrier. Hydrophobins are key contributors to the hydrophobic properties of a wide range of filamentous fungi including ascomycetes and basidiomycetes, suggesting that this is a general function of these proteins (Wösten, 2001). In *Aspergillus oryzae*, hydrophobins interact with cutinases to facilitate efficient degradation of solid polymers (Takahashi et al., 2005). Similar findings in the plant pathogen *Pyricularia oryzae* (syn. *P. grisea, Magnaporthe oryzae* and *M. grisea*) suggest that hydrophobins contribute to the degradation of plant wax layers (Pham et al., 2016). Class I hydrophobin Mpg1 in *P. oryzae* is important for appressorium formation and pathogenicity (Beckerman and Ebbole, 1996; Talbot et al., 1996). The class II hydrophobin Mhp1 of this fungus is also required for virulence (Kim et al., 2005). In *Verticillium dahliae*, class II hydrophobins VDH1 and VdHP1 are required for microsclerotia formation (Klimes et al., 2008; Zhang et al., 2022). Deletion of *VDH1* does not significantly affect virulence, but deletion of *VdHP1* increases crude toxin content and enhances virulence (Zhang et al., 2022). In contrast, hydrophobins are not required for pathogenicity in *Cladosporium fulvum* or *Botrytis cinerea* (Whiteford and Spanu, 2001; Mosbach et al., 2011). These data suggest that the functions of hydrophobins vary among fungal species.

Most filamentous fungi have multiple hydrophobins. The genome of *A. nidulans* is currently known to encodes six, that of *Fusarium graminearum* encodes five, that of *Botrytis cinerea* encodes three, and that of *P. oryzae* encodes two (Talbot et al., 1993; Kim et al., 2005; Mosbach et al., 2011; Grünbacher et al., 2014; Quarantin et al., 2019). In *B. cinerea*, the deletion of at least two hydrophobins leads to aberrant development of apothecia, but the loss of a single one does not affect sexual reproduction (Terhem and van Kan, 2014). These data point to functional redundancy.

The necrotrophic pathogen *Bipolaris maydis* (syn. *Cochliobolus heterostrophus*) causes southern corn leaf blight. Turgeon et al. (2008) reported that the lack of a gene encoding the non-ribosomal peptide synthetase 4 (Nps4) leads to a wettable phenotype (i.e. low hydrophobicity) in this fungus. Colony hydrophobicity is significantly higher in the wild type than in a strain lacking the *CHK1* gene for mitogen-activated protein kinase (MAPK) (Degani et al., 2013). The genome of *B. maydis* is predicted to encode at least four hydrophobins (Degani et al., 2013). These authors used BLAST searches to identify hydrophobins in this fungus but might not have identified all of them due to the low sequence homology among hydrophobins. Furthermore, the effect of the deletion of these genes on morphogenesis and pathogenicity have not been analyzed, and it remains unclear whether hydrophobins contribute to the hydrophobic properties of the cell surface in this fungus.

In this study, to find previously uncharacterized hydrophobins of *B. maydis*, we conducted a comprehensive *in silico* analysis using publicly available amino acid sequence data. To investigate the biological functions of the predicted hydrophobins, we generated single-null mutants as well as mutants combining deletions of the identified genes. We also examined the interspecies compatibility of hydrophobins by heterologous expression of a *B. maydis* hydrophobin in *A. nidulans*.

## 2 Materials and methods

### 2.1 Strains and culture conditions

*Bipolaris maydis* HITO7711 (mating type *MAT1-2*) was used as the wild-type strain throughout this study. Albino strain *alb3* (mating type *MAT1-1*) was used in a crossing assay (Tanaka et al., 1991). The strain lacking the *CHK1* gene (Δ*chk1*) was used as a negative control in a hydrophobicity assay (Izumitsu et al., 2009; Degani et al., 2013). All gene disruptants of *B. maydis* were derived from HITO7711. MASHIKI2-2 (*MAT1-1*) was used as an alternative wild-type strain in a following crossing. For the crossing assay, the quadruple disruptant of hydrophobin genes (*MAT1-1*) was obtained by crossing the quadruple disruptant (*MAT1-2*) and MASHIKI2-2.

*Aspergillus nidulans* ABPU1 strain (Δ*ligD*), which is highly efficient for gene disruption via homologous recombination, was used as a parental strain to obtain a *dewA* gene-disruptant (Hagiwara et al., 2007; Yoshimi et al., 2013). The BPU1 strain carrying the *argB* gene was used as a control (CNT) strain for phenotypic comparisons (Hagiwara et al., 2007; Miyazawa et al., 2021).

All strains were maintained on complete medium agar (CMA; Tanaka et al., 1991) or vegetable juice agar (VJA) medium at 25°C. VJA contained vegetable juice (Kagome, Tokyo, Japan) instead of V8 juice but otherwise had the same composition as V8 agar medium (Ribeiro, 1978); no difference in the growth of *B. maydis* was detected between V8 agar and VJA (data not shown). In the crossing assay, Sachs’s agar medium was used. In a stress tolerance assay, CMA was used. Czapek–Dox (CD) medium was used for culturing *A. nidulans* (Fujioka et al., 2007).

### 2.2 Identification of hydrophobins in *B. maydis*

Hydrophobins are small secreted proteins with a signal peptide and a conserved characteristic C-terminal sequence. To identify the hydrophobins of *B. maydis*, we downloaded the translated sequence from gene catalog of *Cochliobolus heterostrophus* C5 v2.0 from the website of the DOE Joint Genome Institute (JGI, https://mycocosm.jgi.doe.gov/CocheC5_3/CocheC5_3.home.html, Ohm et al., 2012; Condon et al., 2013). In this dataset, we first searched for proteins that were cysteine rich (≥4) and small (≤250 amino acids). Next, we checked whether those proteins possess signal peptides using SignalP 5.0 (https://services.healthtech.dtu.dk/services/SignalP-5.0/). From the refined data, we visually selected proteins with a cysteine pattern specific to hydrophobins (-C-CC-C-C-CC-C-). Additionally, we performed the same search using the updated v3.0 dataset (https://mycocosm.jgi.doe.gov/CocheC5_4m/CocheC5_4m.home.html, Haridas et al., 2023). Hydropathy plots were investigated using ProtScale (https://web.expasy.org/protscale/) based on the Kyte and Doolittle (1982) method. The amino acid sequences of *B. maydis* and *P. oryzae* hydrophobins were aligned with MUSCLE using MEGA X (Edgar, 2004; Kumar et al., 2018). In the alignment and the analysis of the hydropathy plots, the amino acid sequences of *P. oryzae* Mpg1 (class I) and Mhp1 (class II) were used as references. For *in silico* characterization, InterProScan (https://www.ebi.ac.uk/interpro/search/sequence/) was used to analyze each *B. maydis* hydrophobin.

### 2.3 RT-PCR analysis of hydrophobin genes in *B. maydis*

The wild-type strain was incubated under three different conditions: liquid culture, on solid medium, or on a host plant (maize leaves). In liquid culture, the conidia (final concentration, 10^5^ conidia/mL) were added to 100 mL CM liquid medium and incubated with shaking. On solid medium, 100 conidia were inoculated onto cellophane membrane placed on the top of Petri plates filled with CMA. On the host plant, conidial suspension (20 μL of 5.0 × 10^5^ conidia/mL) was inoculated at five points on a single detached 1-month-old leaf. Subsequently, five lesion areas were cut off from leaves and collected. All incubations were carried out at 25°C for 3 days in the dark and were repeated 3 times. Total RNA was extracted from mycelia or leaf lesion areas using Trizol, according to Yoshida and Tanaka (2019). cDNA was synthesized from the total RNA with SuperScript IV reverse transcriptase (Thermo Fisher Scientific, Waltham, MA, USA) using the (dT)21VN primer.

### 2.4 Generation of disruptants of *B. maydis*

The procedure for generating disruptants and locations of primers to confirm gene disruptions are shown in **Supplementary Figure S1A**. All primers are listed in **Supplementary Table S1**. A disruption cassette was constructed for each gene encoding a putative hydrophobin. PCR to construct these cassettes was performed with Takara Ex Taq (Takara Bio, Kusatsu, Japan). All PCR confirmations of gene disruption were performed with SapphireAmp Fast PCR Master Mix (Takara Bio).

HYP1 *disruptant (Δ*hyp1*):* The genomic DNA of *B. maydis* HITO7711 was obtained as described by Izumitsu et al. (2012). The upstream (primer set Hyp1-f1/Hyp-r1) and downstream regions (Hyp-f2/Hyp-r2) of the *HYP1* gene ORF were amplified from genomic DNA. The neomycin phosphotransferase II gene (*NPTII*) cassette was amplified from the pZGenI plasmid (primer set NPTandHPH-f1/NPTII-r1; Sumita et al., 2017). The three amplified products were fused by fusion-PCR (Hyp1-f3/Hyp1-r3 primer set). The resulting cassette was purified by ethanol precipitation and used for *HYP1* disruption. The transformation was based on the PEG-mediated protoplast transformation method described in Izumitsu et al. (2009). The replacement of *HYP1* by the disruption cassette was confirmed by PCR with three primer sets (Hyp1-f1/NPTII-chk1, Hyp1-f4/Hyp1-r4 and NPTandHPH-chk2/Hyp1-r1). A similar approach was used to disrupt *HYP2*–*HYP4* individually and to generate Δ*hyp2*–Δ*hyp4* strains.

*Double, triple, and quadruple disruptants:* To generate a double disruptant, Δ*hyp2*Δ*hyp3*, the *HYP2* gene was replaced in the parental Δ*hyp3* strain using a disruption cassette with a nourseothricin-resistance gene (*NAT*). The *NAT* gene was amplified from the pZNAT1 plasmid with the primer set NATandBAR-f1/NAT-r1 (Izumitsu et al., 2009). To generate the triple disruptant, Δ*hyp2*Δ*hyp3*Δ*hyp4*, the *HYP4* gene of the double disruptant Δ*hyp2*Δ*hyp3* was replaced with the bialaphos-resistance gene (*BAR*) cassette. The *BAR* gene cassette was amplified from the pCB1546 plasmid with the primer set NATandBAR-f1/BAR-r1 (Sweigard et al., 1997). To generate the quadruple disruptant, Δ*hyp1*Δ*hyp2*Δ*hyp3*Δ*hyp4*, the *HYP1* gene of the triple disruptant was replaced with a hygromycin B phosphotransferase gene (*HPH*) cassette. The *HPH* cassette was amplified from the plasmid pCB1004 (Carroll et al., 1994).

### 2.5 Stress tolerance assay

CMA was supplemented with 0.05% SDS, 0.015% H_2_O_2_, or 25 μg/mL Calcofluor White (CFW; Sigma-Aldrich, Saint Louis, MO, USA). Each strain was incubated for 7 days at 25°C, and their colony diameters were measured. To quantify the reduction in radial-growth of colonies, we calculated the percentage decrease relative to the untreated control.

### 2.6 Pathogenicity assay

Maize plants (*Zea mays* cv. Takanestar) were grown at 25°C for 1 month. Freshly detached leaves were placed in plastic cases lined with wet paper towels. Conidia of each strain were harvested from colonies grown on VJA for 7 days. Twenty μL of 5.0 × 10^5^ conidia/mL suspensions (in 0.01% Tween-20) were dropped onto the leaves and incubated in the dark at 25°C for 3 days. We measured and photographed the lesions on the leaves.

### 2.7 Crossing assay

*Bipolaris maydis* forms a characteristic sexual organ called a pseudothecium; usually, it is black in the wild-type strain and light tawny in the albino strain. The ability of any strain to form pseudothecia can be evaluated by using the albino strain in a crossing assay. The crossing assay was performed according to Tanaka et al. (1991). Dried maize leaves were placed on Sachs’s agar medium, then each disruptant (*MAT1-2*) and the albino strain (*MAT1-1*) were inoculated diagonally across the leaves and incubated at 25°C in the dark for 30 days. The pseudothecia were photographed. Both black and light tawny pseudothecia were harvested and dissected in a drop of 10% glycerol on a glass slide to observe the asci and ascospores within the pseudothecia, respectively, under a microscope (Leica DML, Leica Microsystems, Wetzlar, Germany).

### 2.8 Generation of complementation strains of *A. nidulans*

The gene disruption strategies and locations of primers to confirm the insertion are shown in **Supplementary Figure S2**. PCR for plasmid construction was performed with PrimeSTAR GXL DNA polymerase (Takara Bio). All PCR confirmations were performed with Takara Ex Premier DNA Polymerase (Takara Bio). NEBuilder HiFi DNA Assembly Master Mix (New England Biolabs, Ipswich, MA, USA) was used for fusions. Fungal transformations were performed using the PEG-mediated protoplast transformation method as described in Yoshimi et al. (2013).

To construct a disruption cassette of the *A. nidulans dewA* gene (for class I hydrophobin), the upstream and downstream regions of this gene were amplified from genomic DNA with the primer sets dewA-f1/dewA-r1 and dewA-f2/dewA-r2. The *argB* gene of *A. oryzae* (used in this work as selection marker) was amplified from the pAORB plasmid with a primer set AoargB-f1/AoargB-r1 (Furukawa et al., 2002). The three amplicons were fused into a *Bam*HI-linearized pUC19 plasmid. The *dewA* gene disruption cassette was amplified from the resulting plasmid with the primer set pUC-chk1/pUC-chk2, purified by ethanol precipitation and integrated into the ABPU1 (*ligD*) strain. The candidate disruptants were inoculated and incubated on selective CD medium without arginine. The replacement of the *dewA* gene with the *argB* gene was confirmed with the dewA-f3/AoargB-chk1, dewA-f4/dewA-r4, and AoargB-chk2/dewA-r3 primer sets.

For complementation assays with the *dewA* and *B. maydis* hydrophobin genes, we constructed a plasmid to reintroduce the *dewA* gene and four plasmids to introduce *B. maydis* hydrophobin genes into the Δ*dewA* strain. To generate the pUC-pyrG plasmid, the entire *A. nidulans pyrG* gene with the putative promoter was amplified with the primer set pyrG-f1/pyrG-r1, and the PCR fragment was fused into a *Bam*HI-linearized pUC19 vector. To generate the *dewA* complementation plasmid, the entire *dewA* gene with the promoter and ORF was amplified with the primer set dewA-pyrG-f1/dewA-pyrG-r1 and fused into a *Sma*I-linearized pUC-pyrG plasmid.

To generate the *HYP1* complementation plasmid, the *HYP1* gene with the 3′ untranslated region from the start codon was amplified from *B. maydis* genomic DNA with the primer set HYP1-start-f1/HYP1-pyrG-r1. The putative promoter region (ca. 1000 bp upstream of *dewA* ORF) was amplified with the primer set dewA-pyrG-f1/dewA-HYP1-r1. These two PCR fragments were fused into the *Sma*I-linearized pUC19-pyrG plasmid. Complementation plasmids for the remaining three *B. maydis* hydrophobins were constructed in a similar fashion.

To generate *dewA*^comp^ and *B. maydis* hydrophobin-complemented strains (*HYP1*–*4*^comp^), the corresponding complementation plasmids were introduced into the Δ*dewA* strain. Resulting candidate strains were inoculated and incubated on selective CD medium without arginine, uridine, and uracil. Insertion of each gene was confirmed with the corresponding primer set f4/r4.

### 2.9 RT-PCR analysis of hydrophobin genes in *A. nidulans*

The CNT, Δ*dewA*, *dewA*^comp^, and *HYP1*–*4*^comp^ strains were incubated on CD medium for 1 week. Conidia (final concentration, 10^5^ conidia/mL) from each colony were added to 20 mL of CD liquid medium and incubated with shaking for 24 hours. The extraction of total RNA from the mycelia and the synthesis of cDNA were performed as described above. The histone H2B gene was used as internal control (Yoshimi et al., 2013).

### 2.10 Hydrophobicity assays in *B. maydis* and *A. nidulans*

Colonies of the wild-type strain and all disruptants of *B. maydis* were grown on CMA at 25°C for 7 days. The colonies were punched 1 cm from their edge with a cork borer (diameter: 1 cm), and 10 µL of a solution containing 0.2% SDS and 50 mM ethylenediaminetetraacetic acid (EDTA) was dropped onto the mycelial disks. We took photos of these droplets every 10 min for 30 min to record their changes.

CNT, Δ*dewA*, *dewA*^comp^, and *HYP1*–*4*^comp^ strains of *A. nidulans* were incubated on CD medium at 37°C for 4 days. The colonies were punched at the edge using a cork borer, aligning the borer’s perimeter with the colony’s perimeter. The hydrophobicity of each strain was assessed as above. Photographs were taken every minute for 5 min.

## 3 Results

### 3.1 Four hydrophobins in *B. maydis*

**Figure 1:**
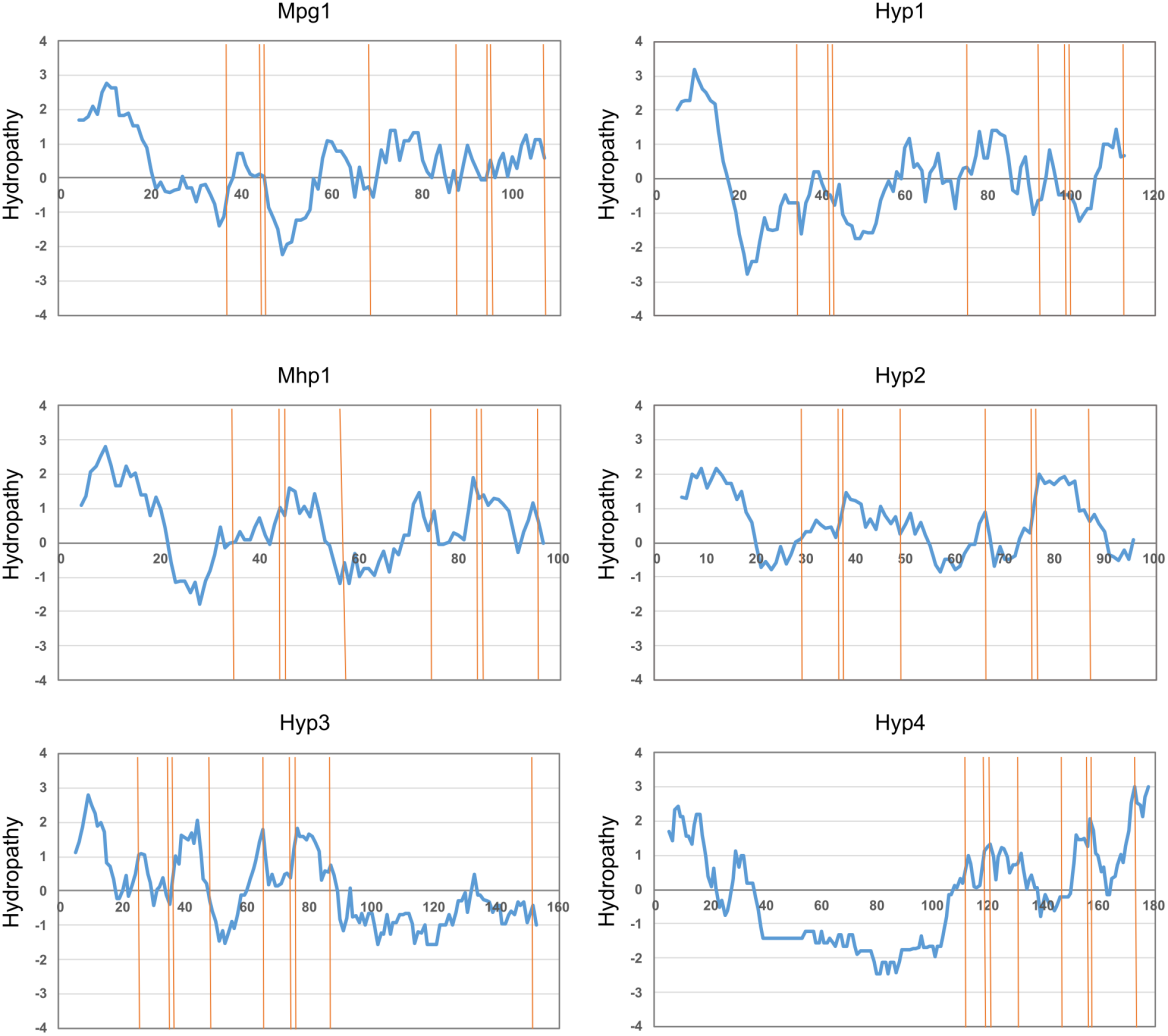
Hydropathy plots of *B. maydis* hydrophobins. Hydropathy was calculated using ProtScale with the method of Kyte and Doolittle (1982). Hydrophobins of *P. oryzae* Mpg1 (class I) and Mhp1 (class II) were used as controls.

By searching the gene catalog (protein data) of *C. heterostrophus* C5 from the JGI website, we identified four candidate proteins with a signal peptide and a cysteine pattern characteristic of hydrophobins. The total length was 117 amino acids for Hyp1, 100 amino acids for Hyp2, 157 amino acids for Hyp3, and 182 amino acids for Hyp4. Each of the hydrophobin genes had two exons. The hydropathy profile of Hyp1 was similar to that of Mpg1 of *P. oryzae* while that of Hyp2 was similar to that of Mhp1 (**Figure 1**; **Supplementary Figure S3**). Those of Hyp3 and Hyp4 were also similar to that of Mhp1, except some regions (**Figure 1**; **Supplementary Figure S3B**). Hyp3 had a unique long sequence (71 amino acids) after the 8th cysteine, which contained a 9th cysteine (**Supplementary Figure S3B**). Hyp4 had 90 amino acids, approximately half of them glycines, between the signal peptide and the first cysteine (**Supplementary Figure S3B**). InterProScan searches showed that Hyp1 belongs to the hydrophobin family (InterPro IRR001338; class I), whereas Hyp2–Hyp4 belong to the cerato-ulmin hydrophobin family (InterPro IRR010636; class II). Cerato-ulmin is a representative class II hydrophobin produced by *Ophiostoma ulmi* (Stringer and Timberlake, 1993; Sbrana et al., 2010).

### 3.2 Expression of the four hydrophobin genes

**Figure 2:**
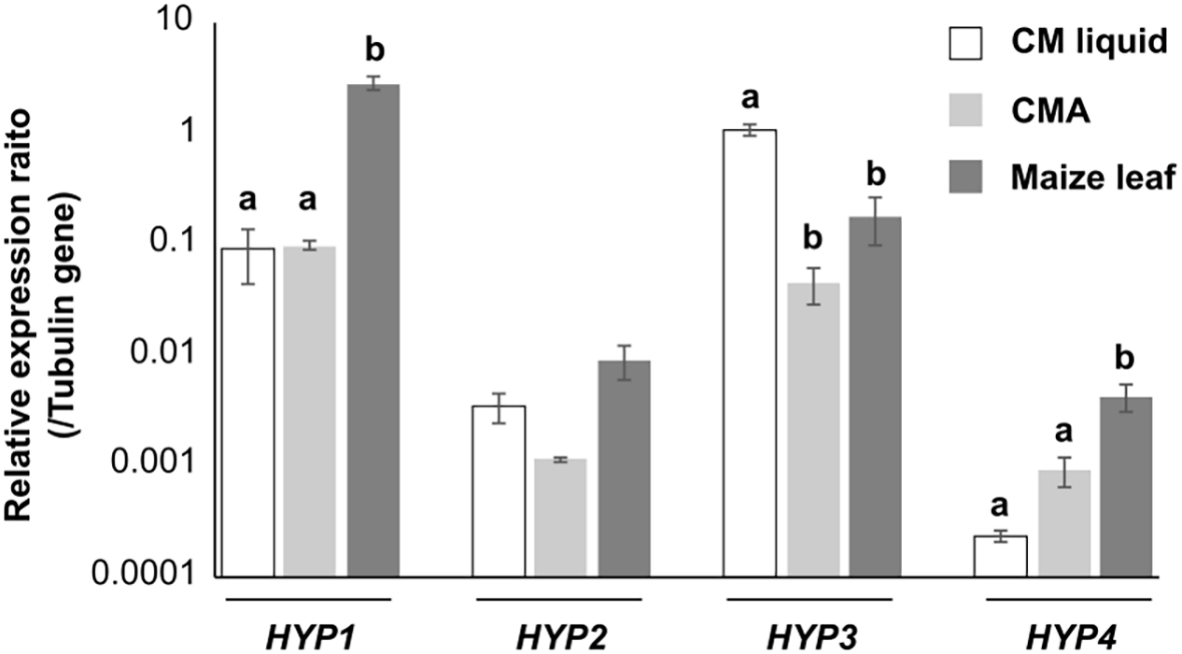
Transcript levels of *B. maydis* hydrophobin genes. Hyphae were cultured for 3 days in CM liquid, on cellophane membranes on CMA, or on the leaves of host plants (maize). RNA was extracted and used for qRT-PCR. Error bars, standard error (*n* = 3). Different letters within each gene indicate significant differences (Tukey’s test, *P* < 0.05).

In mycelia of *B. maydis* incubated in liquid culture, on solid medium, or on maize leaves, all hydrophobin genes were expressed, but at different levels: the transcript levels were higher for *HYP1* and *HYP3* than for *HYP2* and *HYP4* (**Figure 2**), suggesting that this fungus uses mainly Hyp1 and Hyp3. The transcript levels of *HYP1* in maize leaves and *HYP3* gene in liquid culture were similar to that of β tubulin-encoding gene as an internal control. The transcript level of *HYP4* was highest in maize leaves, but that of *HYP2* did not differ significantly among the three conditions. The transcript levels of all hydrophobin genes, except for *HYP2*, differed significantly among the culture conditions, suggesting that each hydrophobin may be used differently under different conditions.

### 3.3 Generation of hydrophobin gene disruptants in *B. maydis*

**Figure 3:**
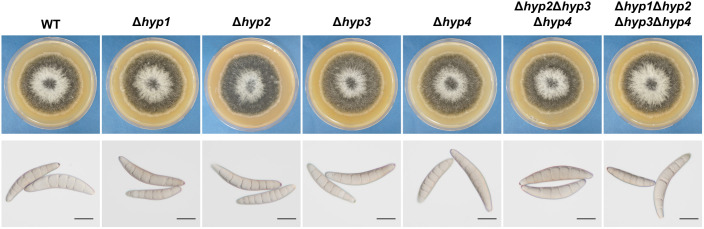
Colony growth and conidial morphology of the wild-type strain and disruptants of hydrophobin genes. Each strain was cultured on VJA for 7 days. Conidia were harvested from three colonies of each strain. Bars: 20 µm.

The single disruption of each hydrophobin gene in *B. maydis* was confirmed by PCR (**Supplementary Figures S1C–F**). A class II hydrophobin-deficient strain (*hyp2*Δ*hyp3*Δ*hyp4*) and a strain deficient in all four hydrophobins (Δ*hyp1*Δ*hyp2*Δ*hyp3*Δ*hyp4*) were also generated (**Supplementary Figure S1A, B, G–I**). All strains were inoculated on VJA and incubated at 25°C for 7 days. Radial growth was similar in all the disruptants and the wild-type strain (**Figure 3**; **Table 1**). Conidia of all strains were harvested from these colonies and analyzed. The conidia of the wild-type strain were spindle-shaped and composed of cells divided by multiple septa. No considerable differences in conidial morphology or the number of conidia were observed between the disruptants and the wild-type strain (**Figure 3**, **Table 1**). These results indicate that the loss of hydrophobins in this fungus does not affect normal radial growth and conidiation.

**Table 1:** Phenotypes of disruptants of hydrophobin genes of *B. maydis*: growth, conidiation, and pathogenicity.

Strain	Colony diam. (mm^2^)^a^	No. of conidia/cm^2^× (×10^4^)	Lesion size 1 (mm)^b^	Lesion size 2 (mm)^b^
Wild type	61.07 ± 0.7	5.9 ± 1.1	13.8 ± 1.3	13.6 ± 0.9
Δ*hyp1*	58.28 ± 2.9	7.2 ± 0.4	13.4 ± 1.4	Not tested
Δ*hyp2*	62.09 ± 0.4	5.2 ± 0.7	13.3 ± 1.3	Not tested
Δ*hyp3*	61.47 ± 1.6	7.1 ± 1.2	14.3 ± 1.2	Not tested
Δ*hyp4*	63.04 ± 0.9	6.1 ± 1.1	14.5 ± 1.4	Not tested
Δ*hyp2*Δ*hyp3* Δ*hyp4*	63.40 ± 0.6	5.8 ± 0.2	Not tested	13.9 ± 0.9
Δ*hyp1*Δ*hyp2* Δ*hyp3*Δ*hyp4*	61.23 ± 0.5	7.3 ± 0.9	Not tested	13.6 ± 0.9

Values are mean ± standard error (colony diameter and conidiation, *n* = 3; lesion size, *n* = 5). Tukey’s test was used to determine statistical significance (*P* < 0.05).

a These colonies were incubated for 7 days at 25°C.

b Lesion size 1: wild type and single disruptants (**Figure 5A**). Lesion size 2: wild type, Δ*hyp2*Δ*hyp3*Δ*hyp4*, and Δ*hyp1*Δ*hyp2*Δ*hyp3*Δ*hyp4* strains (**Figure 5B**).

### 3.4 Stress tolerance of hydrophobin gene disruptants

**Figure 4:**
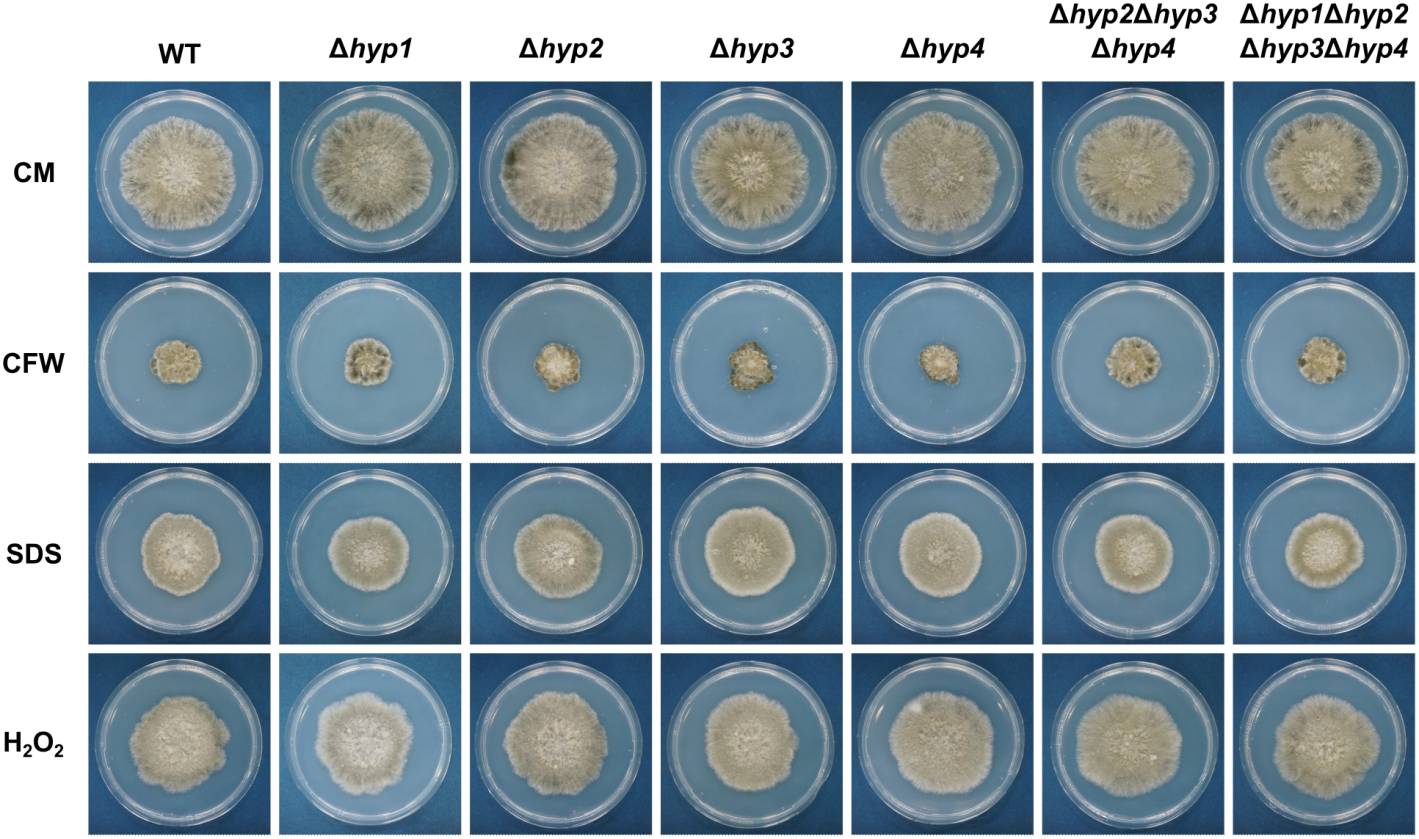
Sensitivity of the wild-type and hydrophobin gene disruptants to stress agents. All strains were incubated on CMA supplemented with 25 μg/mL calcofluor white (CFW), 0.05% SDS, or 0.015% H_2_O_2_ for 7 days.

All strains were cultured on CMA in the absence or presence of stress-inducing compounds (CFW, NaOH, or SDS). The decrease in radial growth of all disruptants on each medium was not significantly different from those of the wild-type strain (**Figure 4**; **Supplementary Figure S4**). These results showed that hydrophobins in *B. maydis* do not contribute to these types of stress tolerance.

### 3.5 Pathogenicity of hydrophobin gene disruptants

**Figure 5:**
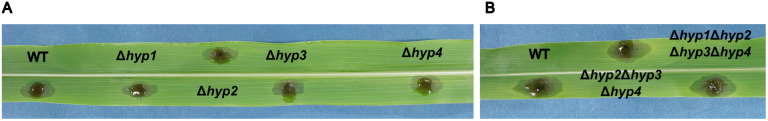
Pathogenicity assay on maize leaves. **(A)** The wild-type strain and single disruptants of each hydrophobin gene. **(B)** The wild-type strain, triple and quadruple disruptants of hydrophobin genes. Photos were taken 3 days after inoculation on maize leaves.

To investigate whether *B. maydis* hydrophobins are involved in pathogenicity, we performed a pathogenicity assay on maize leaves. Lesions formed by each single disruptant were similar to those formed by the wild-type strain (**Figure 5A**; **Table 1**). Similarly, no significant difference was observed in the size of lesions among the triple disruptant, quadruple disruptant, and the wild-type strain (**Figure 5B**; **Table 1**). These results show that the lack of hydrophobins does not affect the pathogenicity of *B. maydis*.

### 3.6 Sexual reproduction of hydrophobin gene disruptants

**Figure 6:**
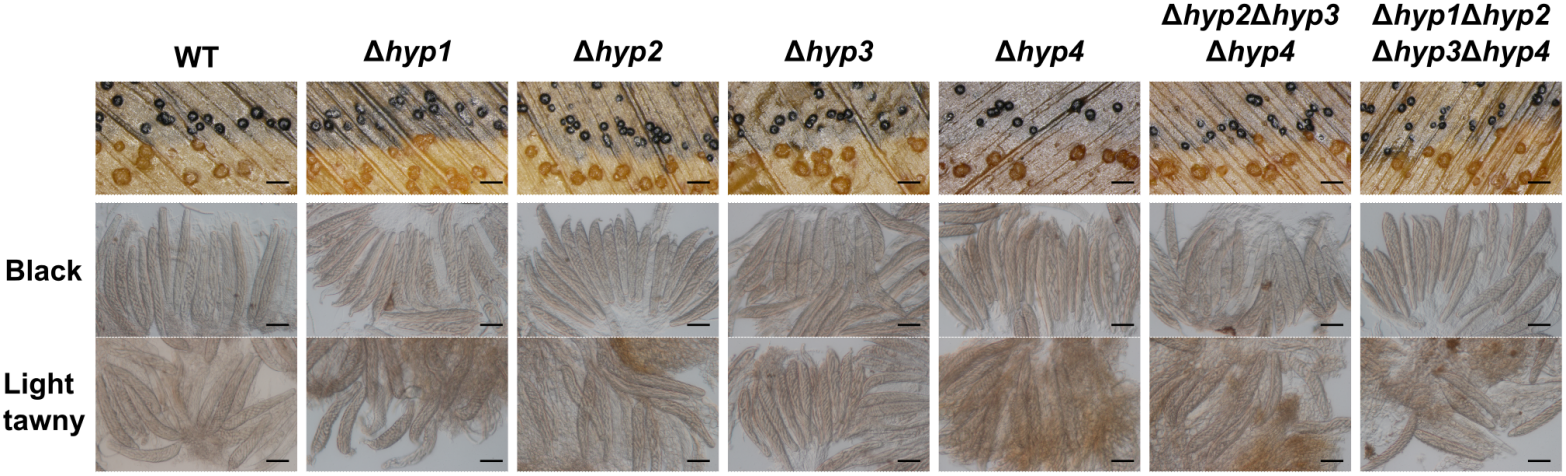
Sexual reproduction of disruptants of hydrophobin genes. The wild-type and each mutant were crossed with an albino strain. Black and light tawny pseudothecia were harvested 30 days after the cross and dissected to observe asci and ascospores. Bars: 1 mm (top), 50 µm (middle and bottom).

In the cross between the wild-type and albino strains, black pseudothecia and light tawny pseudothecia were formed along their contact line (**Figure 6**). When all disruptants were crossed with the albino strain, pseudothecia of black and light tawny colors were formed; therefore, the loss of hydrophobins did not affect the formation of female organs (**Figure 6**). To investigate whether hydrophobins are involved in ascus and ascospore development, pseudothecia of both colors were harvested and inspected. In all crosses with the albino strain, asci containing ascospores were formed in pseudothecia of both colors (**Figure 6**). When the quadruple disruptant was crossed with itself, asci containing ascospores were formed normally in pseudothecia (**Supplementary Figure S5**). These results suggested that hydrophobins in *B. maydis* are dispensable for sexual reproduction.

### 3.7 Colony hydrophobicity of hydrophobin gene disruptants

**Figure 7:**
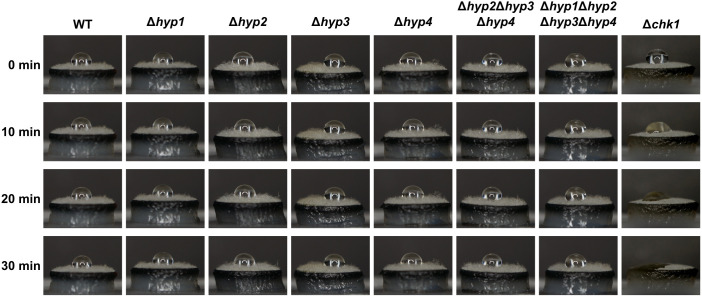
Hydrophobicity of the wild type and disruptants of hydrophobin genes. A 10-μL droplet of solution containing 0.2% sodium dodecyl sulfate and 50 mM ethylenediaminetetraacetic acid was placed on the mycelial disk of each strain and observed at the indicated time points.

To investigate whether hydrophobins play a role in *B. maydis* colony hydrophobicity, solution containing 0.2% SDS and 50 mM EDTA was dropped on mycelial disks (**Figure 7**). The Δ*chk1* strain, disruptant of the *CHK1* gene encoding MAPK, was used as the control because its colonies are significantly less hydrophobic than those of the wild type (Degani et al., 2013). The droplet on the mycelial disk of Δ*chk1* strain changed its shape after 10 min and disappeared completely after 20 min. The droplet on the wild-type strain remained unchanged after 30 min. In all disruptants, including the quadruple one, droplets also remained unchanged after 30 min (**Figure 7**). These results showed that *B. maydis* hydrophobins are not required for colony hydrophobicity.

### 3.8 Colony hydrophobicity of *dewA* gene disruptant complemented with *B. maydis hydrophobin*

**Figure 8:**
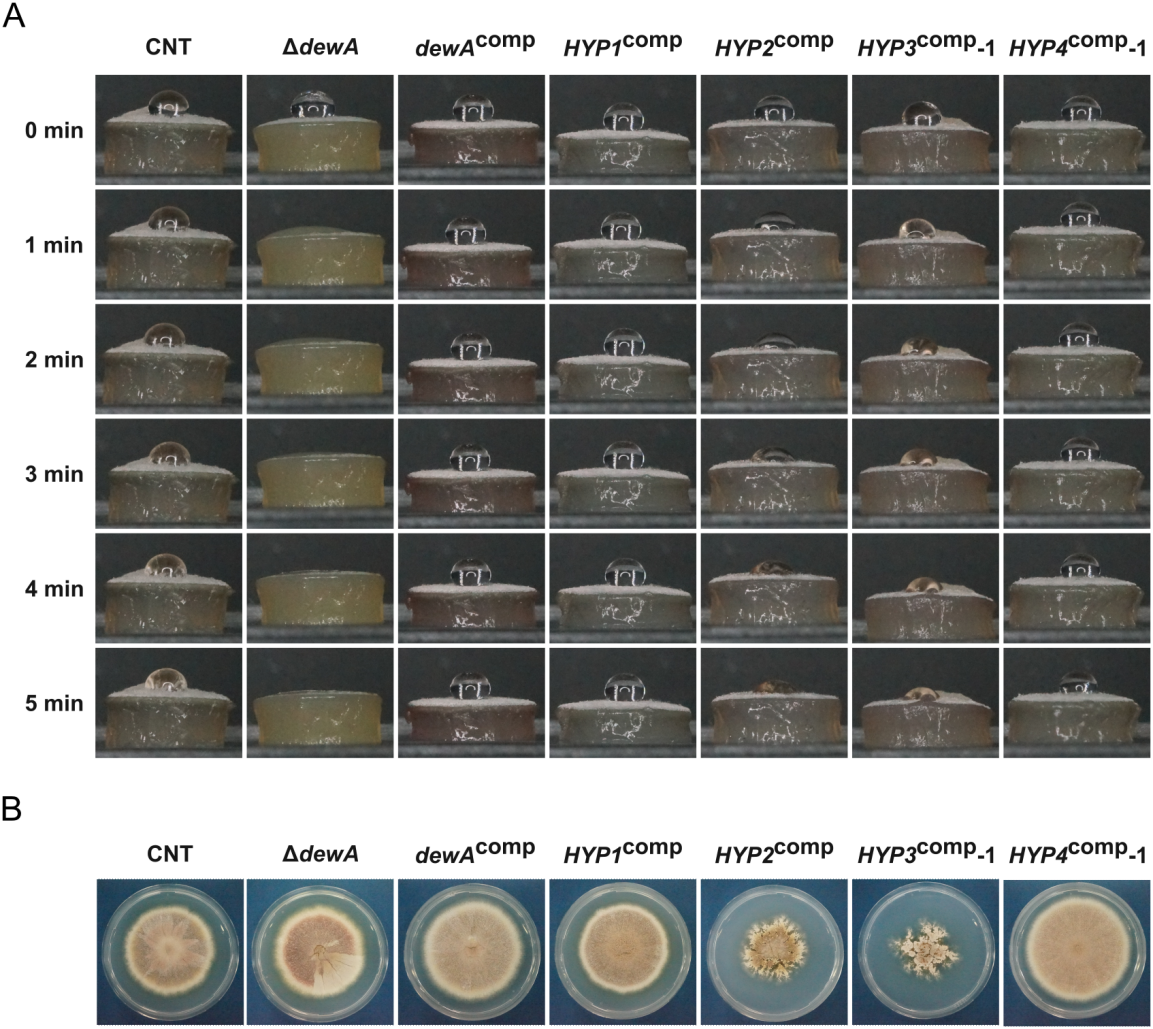
Colony phenotype of *A. nidulans* mutants: CNT, Δ*dewA*, *dewA*^comp^, and Δ*dewA* with *B. maydis* hydrophobins. **(A)** Colony growth. Each strain was incubated on CD medium for 1 week. **(B)** Hydrophobicity of each strain. A 10-μL droplet of solution containing 0.2% sodium dodecyl sulfate and 50 mM ethylenediaminetetraacetic acid was placed on the mycelial disk of each strain and observed at the indicated time points.

From the above results, we hypothesized that the hydrophobins of *B. maydis* lost their ability to provide hydrophobicity. To test this hypothesis, we investigated whether *B. maydis* hydrophobin could restore the hydrophobicity of a hydrophobin-deficient mutant of *A. nidulans*. *Aspergillus nidulans* with a disruption of the *dewA* gene encoding a class I hydrophobin shows significant mycelial wettability (Grünbacher et al., 2014). First, we generated a *dewA* gene disruptant strain (**Supplementary Figure S2A, B**). The droplet on the CNT strain remained unchanged after 5 min, but the droplet on the Δ*dewA* strain disappeared completely after 2 min (**Figure 8A**). This result is consistent with previous studies (Stringer and Timberlake, 1995; Grünbacher et al., 2014). Next, we generated the *dewA*^comp^ strain by reintroducing the wild-type *dewA* gene into the Δ*dewA* strain and confirmed the reintroduction by PCR (**Supplementary Figure S2C, D**). The hydrophobicity of the *dewA*^comp^ strain was similar to that of the CNT strain (**Figure 8A**).

We also constructed complemented strains, in which the *B. maydis* hydrophobin genes under the control of the *dewA* promoter were integrated into the Δ*dewA* strain, and obtained one strain of *HYP1*^comp^ and *HYP2*^comp^ each, three *HYP3*^comp^ strains and two *HYP4*^comp^ strains (**Supplementary Figure S2C, D**). While the colony growth of the *HYP1*^comp^ and *HYP4*^comp^ strains was similar to that of the CNT strain, *HYP2*^comp^ and *HYP3*^comp^ strains showed slower colony growth and an abnormal colony morphology compared to the CNT strain (**Figure 8B**; **Supplementary Figure S6A**). The expression analysis revealed that *B. maydis* hydrophobin expression in each complemented strain was equivalent to or even exceeded the expression level of *dewA* gene in the CNT strain (**Supplementary Figure S6B**). Similarly to the *dewA*^comp^ strain, the *HYP1*^comp^ strain, complemented with class I hydrophobin *HYP1* gene, regained its colony hydrophobicity (**Figure 8A**). Interestingly, although the degree of hydrophobicity varied, the class II hydrophobin-complemented strains (*HYP2*^comp^, *HYP3*^comp^, and *HYP4*^comp^) also showed increased hydrophobicity compared to the Δ*dewA* strain (**Figure 8A**; **Supplementary Figure S6C**). This result showed that the hydrophobins of *B. maydis* have not lost their ability to confer colony hydrophobicity.

## 4 Discussion

Previous studies in ascomycetes and basidiomycetes show that many filamentous fungi have varying numbers of genes encoding hydrophobins. In these and other fungi, hydrophobins are involved in a variety of functions. In *P. oryzae*, the hydrophobins Mpg1 and Mhp1 are involved in appressorium formation and pathogenicity (Talbot et al., 1996; Kim et al., 2005). RodA on the conidia of *A. fumigatus* masks the cell wall components from recognition by the host immune system (Aimanianda et al., 2009). In *A. oryzae*, RolA interacts with the cutinase CutL1 and is involved in efficient polybutylene succinate-*co*-adipate degradation (Takahashi et al., 2005). In the basidiomycete *Pleurotus ostreatus*, hydrophobin is important for efficient lignin degradation and resistance to environmental stress (Han et al., 2023a, b). However, information about the hydrophobins of the pathogen *B. maydis* has been limited. In this study, we found one class I (Hyp1) and three class II hydrophobins (Hyp2–Hyp4). These number and types of hydrophobins identified in our study are consistent with the findings of Degani et al. (2013), who searched for hydrophobin genes in this fungus using a different method.

We began functional analyses by generating single-gene disruptants for each of the four hydrophobin genes. Each disruptant showed no significant differences compared to the wild-type strain in terms of hyphal growth, conidiation, environmental stress tolerance, virulence, or sexual reproduction (**Figures 3**-**6**). Disruption of a single gene out of several does not always alter the phenotype, so phenotypic analyses using a disruptant of multiple genes are also important. In *B. cinerea*, some double and triple disruptants, but not single disruptants, have abnormal apothecia (Terhem and van Kan, 2014). In *F. graminearum*, the disruption of one to five hydrophobin genes affects pathogenicity, conidiation, and secondary metabolism (Shin et al., 2022).

Here, we generated triple and quadruple disruptants. However, contrary to the reports for other fungi, the phenotype of these strains was similar to those of the wild-type strain and single disruptants. Thus, unlike in some other filamentous fungi, including ascomycetes and basidiomycetes, the hydrophobins of *B. maydis* appear not to play an important role in biological processes.

In general, hydrophobins are thought to be secreted into the extracellular matrix and to confer hydrophobic properties to mycelia and conidia (Wösten, 2001). The lack of class I hydrophobins DewA and RodA reduces colony hydrophobicity in *A. nidulans* (Grünbacher et al., 2014). Hydrophobins are also involved in colony hydrophobicity in several phytopathogenic filamentous fungi including *P. oryzae* and *B. cinerea* (Talbot et al., 1993; Kim et al., 2005; Mosbach et al., 2011). In our study, however, colony hydrophobicity did not differ significantly between the wild-type strain, single disruptants, or the quadruple disruptant (**Figure 7**). These results show that hydrophobins of this fungus are not essential for colony hydrophobicity. In *B. maydis*, hydrophobicity depends on other factors such as the Nps4, as the loss of the *NPS4* gene reduces colony hydrophobicity (Turgeon et al., 2008). These authors attributed colony hydrophobicity to either Nps4 or to the regulation of hydrophobins by it. In conjunction with the present study, it is more likely that the former possibility is correct. Since the disruptant of the *CHK1* gene encoding MAPK had hydrophilic colonies (**Figure 7**; Degani et al., 2013), Nps4 may function downstream of the MAPK pathway. To investigate the function of *B. maydis* hydrophobins in more detail, we introduced the *HYP1, HYP2, HYP3, and HYP4* genes into the Δ*dewA* strain of *A. nidulans*.

Interestingly, all resulting complemented strains showed increased colony hydrophobicity compared to the Δ*dewA* strain (**Figure 8A**; **Supplementary Figure S6C**). Generally, class II hydrophobins possess lower hydrophobicity compared to class I hydrophobins (Wessels et al., 1991). Reflecting this fact, the hydrophobicity of the *HYP1*^comp^ strain was significantly higher compared to that of the *HYP2*^comp^ and *HYP3*^comp^ strains (**Figure 8A**; **Supplementary Figure S6C**). However, the *HYP4*^comp^ strain, despite being a class II hydrophobin, showed similar hydrophobicity to the *HYP1*^comp^ and *dewA*^comp^ strains (**Figure 8A**; **Supplementary Figure S6C**). Furthermore, the expression level of the *HYP4* gene in this strain was significantly higher than that of the *dewA* gene in the CNT and *dewA*^comp^ strains (**Supplementary Figure S6B**). This result suggests that, when their expression level is sufficiently high, class II hydrophobins can confer colonies hydrophobic properties comparable to class I hydrophobins. Although *A. nidulans* does not possess any endogenous class II hydrophobins (Tanaka et al., 2022), these results showed that exogenous class II hydrophobins are functionally viable in this fungus. Considering that *HYP2*^comp^ and *HYP3*^comp^ strains formed abnormal colony morphologies (**Figure 8B**; **Supplementary Figure S6A**), it is possible that the secretion process of the exogenous class II hydrophobins, or their behavior within the extracellular matrix causes some disadvantages to the growth of this fungus. Furthermore, unlike Hyp2 and Hyp3, the presence of the characteristic Gly-rich sequence at the N-terminus of Hyp4 may play a role in mitigating these adverse effects, explaining the normal colony morphology of the *HYP4*^comp^ strain, despite it being a class II hydrophobin (**Figure 1**; **Supplementary Figure S3**). These findings strongly suggest that hydrophobins possess different functional roles depending on the fungal species.

We were unable to detect any phenotypic changes in the quadruple disruptant of hydrophobin genes in *B. maydis*. However, we found that the four genes encoding hydrophobins in this fungus are expressed, and that all four hydrophobins have retained their function as a hydrophobin when expressed in *A. nidulans*. Hence, these hydrophobins may have some functionally redundant roles in *B. maydis* or their role may not be the conferral of colony hydrophobicity. To clarify what their role is in *B. maydis*, it is necessary to analyze the hydrophobin proteins, including their localization in this fungus. Analyses of the regulation of hydrophobins in these two fungal species with different mechanisms of colony hydrophobicity may shed light on the diversification of hydrophobin function.

## Data Availability

The original contributions presented in the study are included in the article/**Supplementary Material**. Further inquiries can be directed to the corresponding author.
