# Supplementary figures and images for: Hydrophobins in Bipolaris maydis do not contribute to colony hydrophobicity, but their heterologous expressions alter colony hydrophobicity in Aspergillus nidulans
Source: Front Fungal Biol. 2025 Nov 20;6:1604903. doi: 10.3389/ffunb.2025.1604903 (PMC12675215; doi:10.3389/ffunb.2025.1604903)

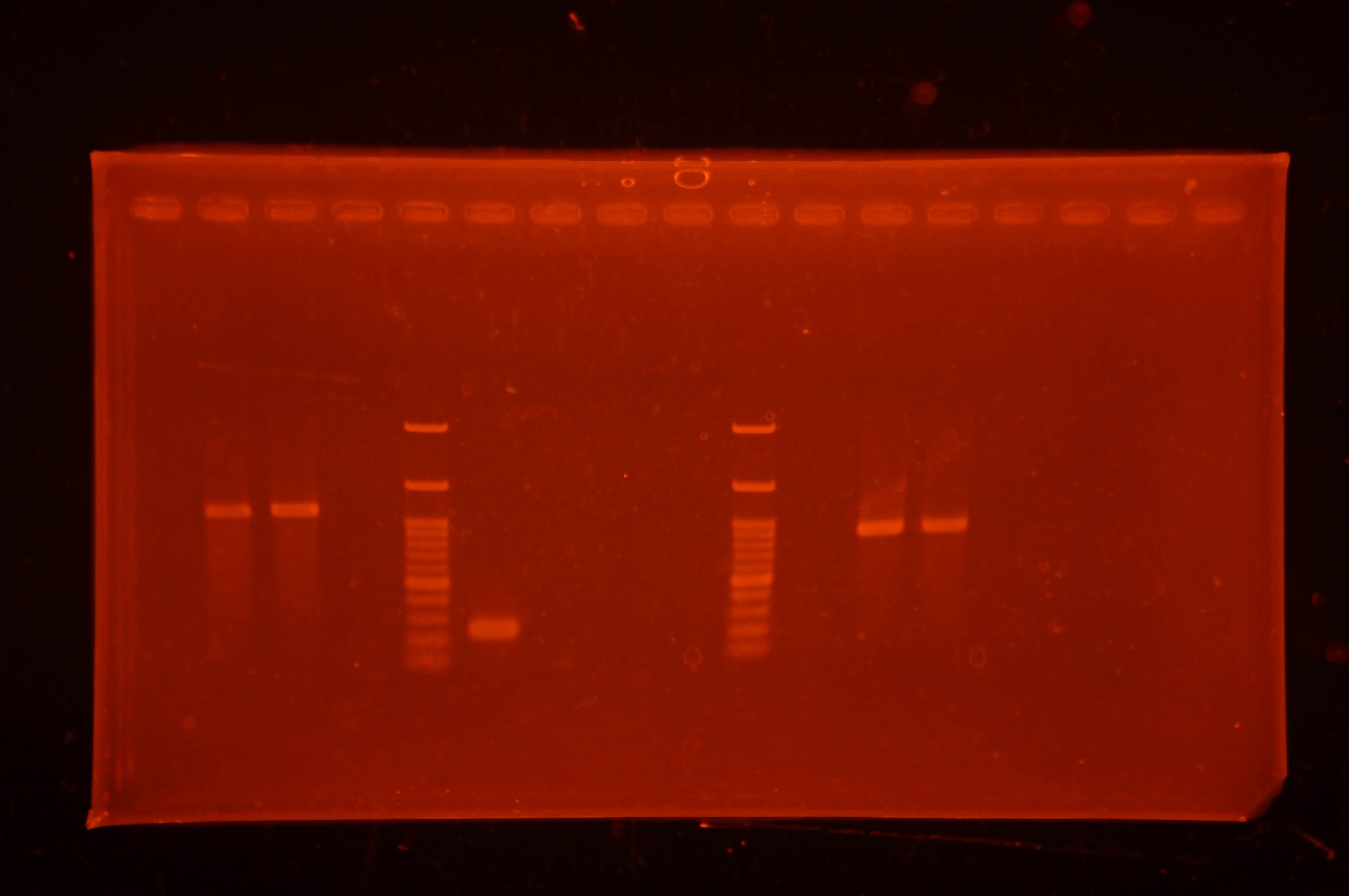

Supplement: Supplementary file 1 [file Image1.jpeg]

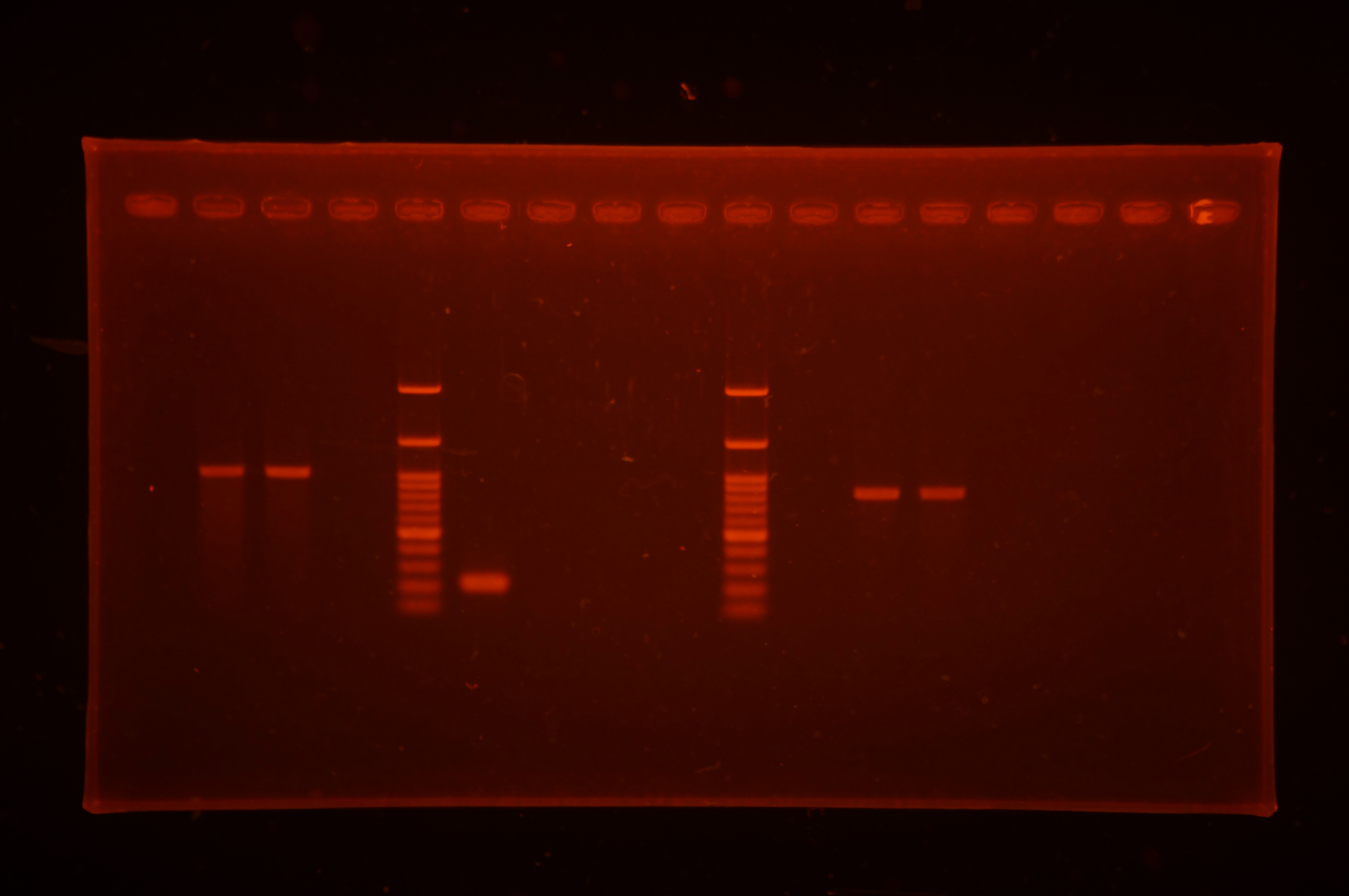

Supplement: Supplementary file 2 [file Image2.jpeg]

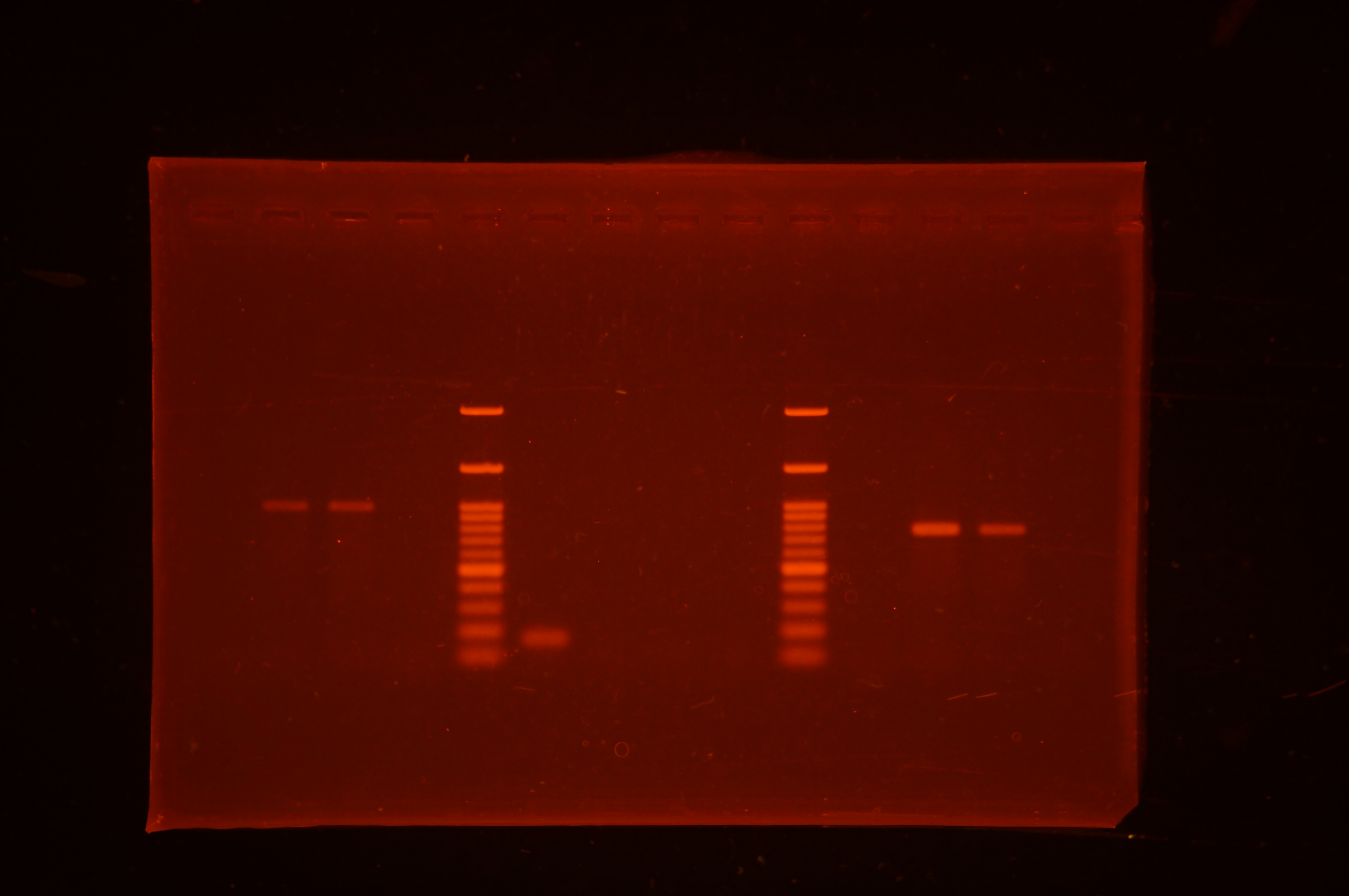

Supplement: Supplementary file 3 [file Image3.jpeg]

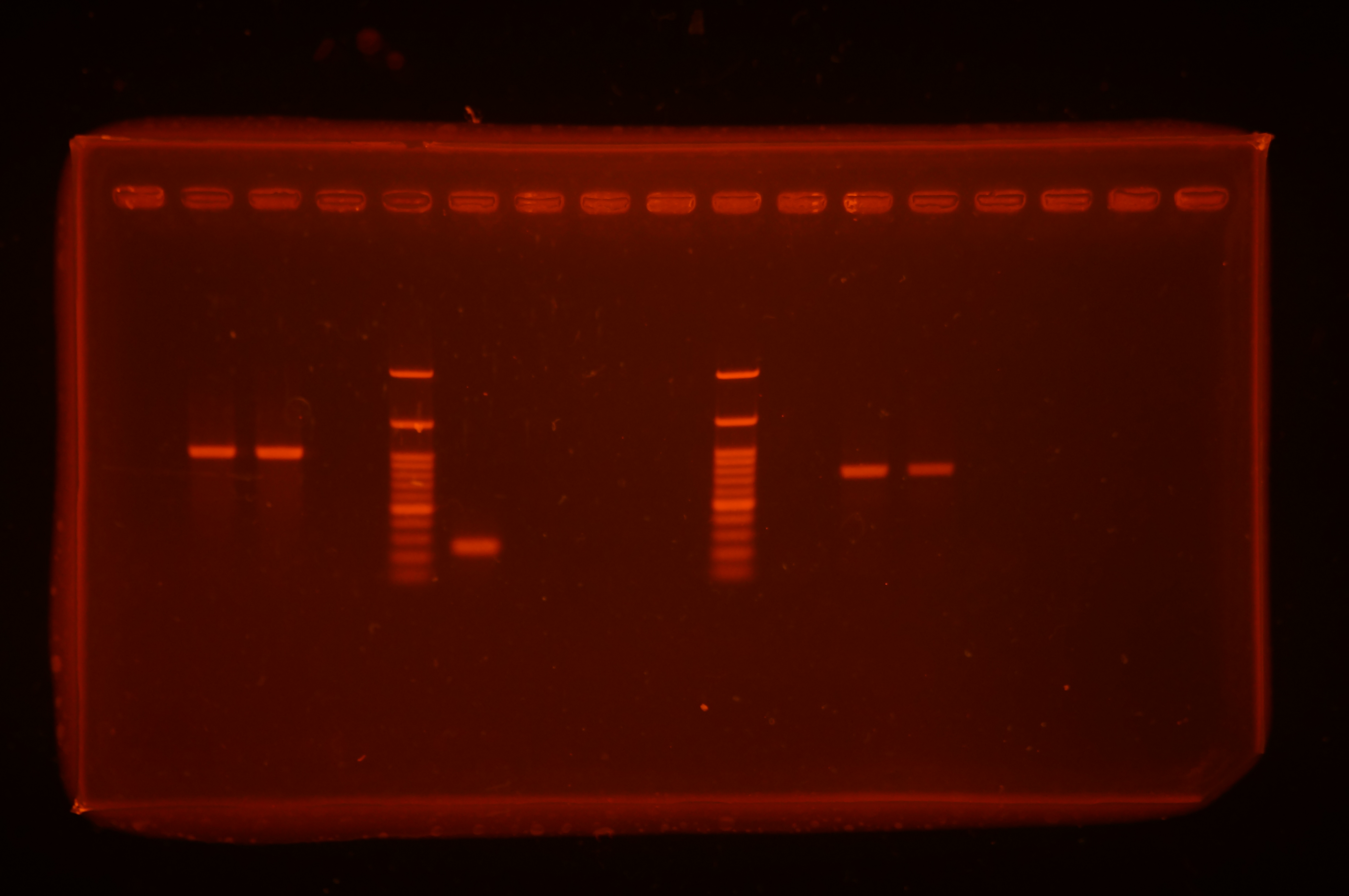

Supplement: Supplementary file 4 [file Image4.jpeg]

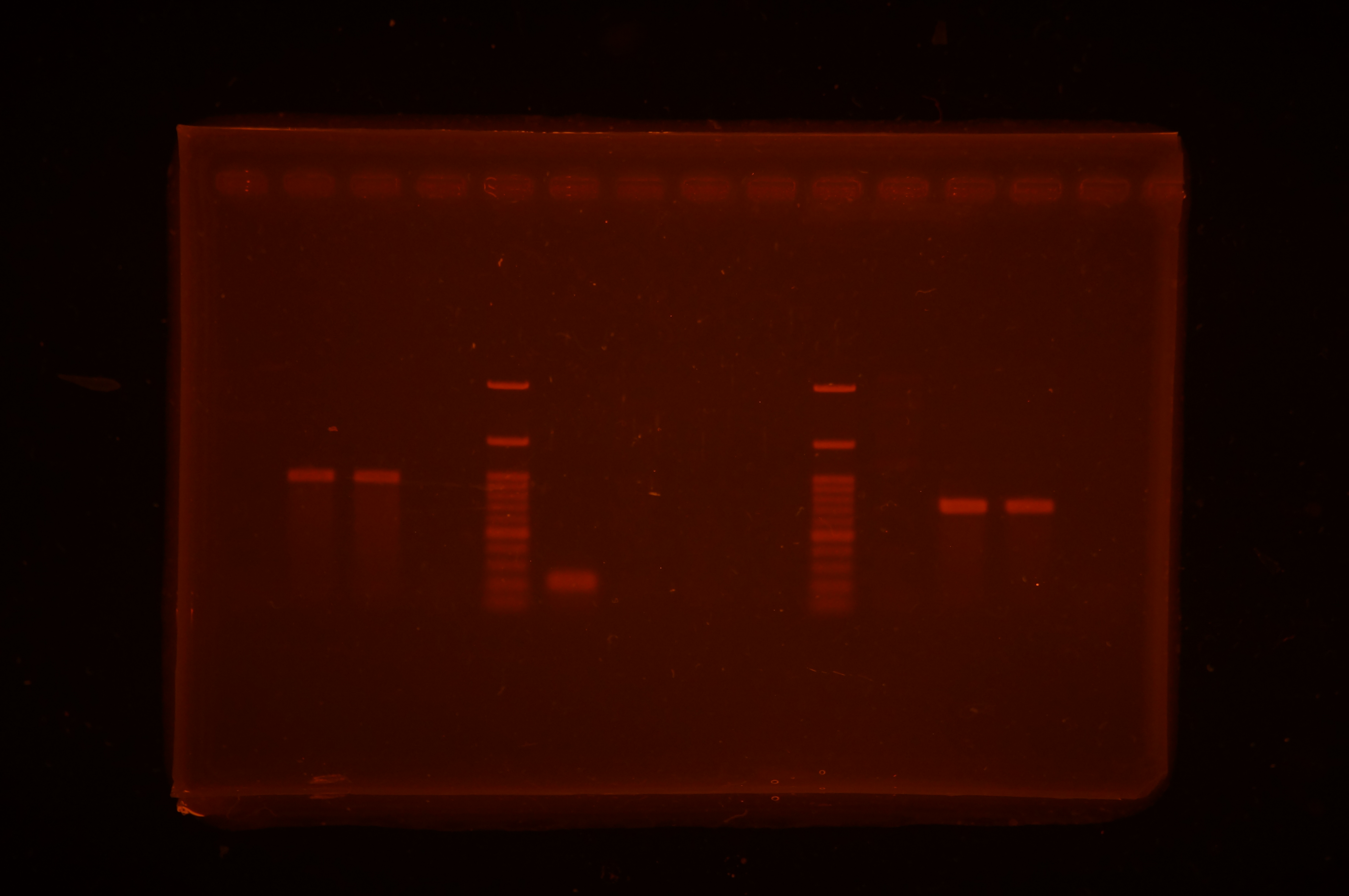

Supplement: Supplementary file 5 [file Image5.jpeg]

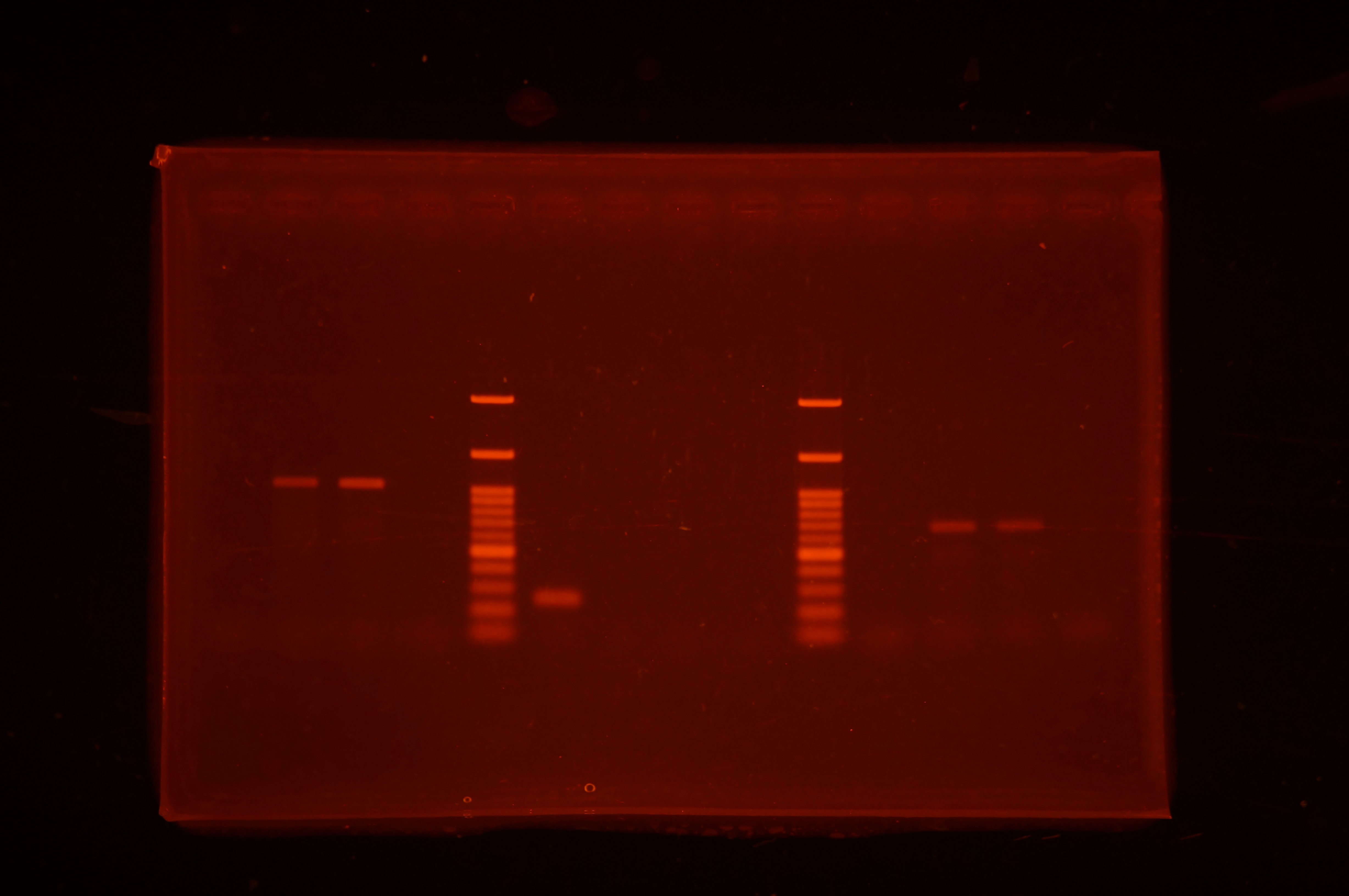

Supplement: Supplementary file 6 [file Image6.jpeg]

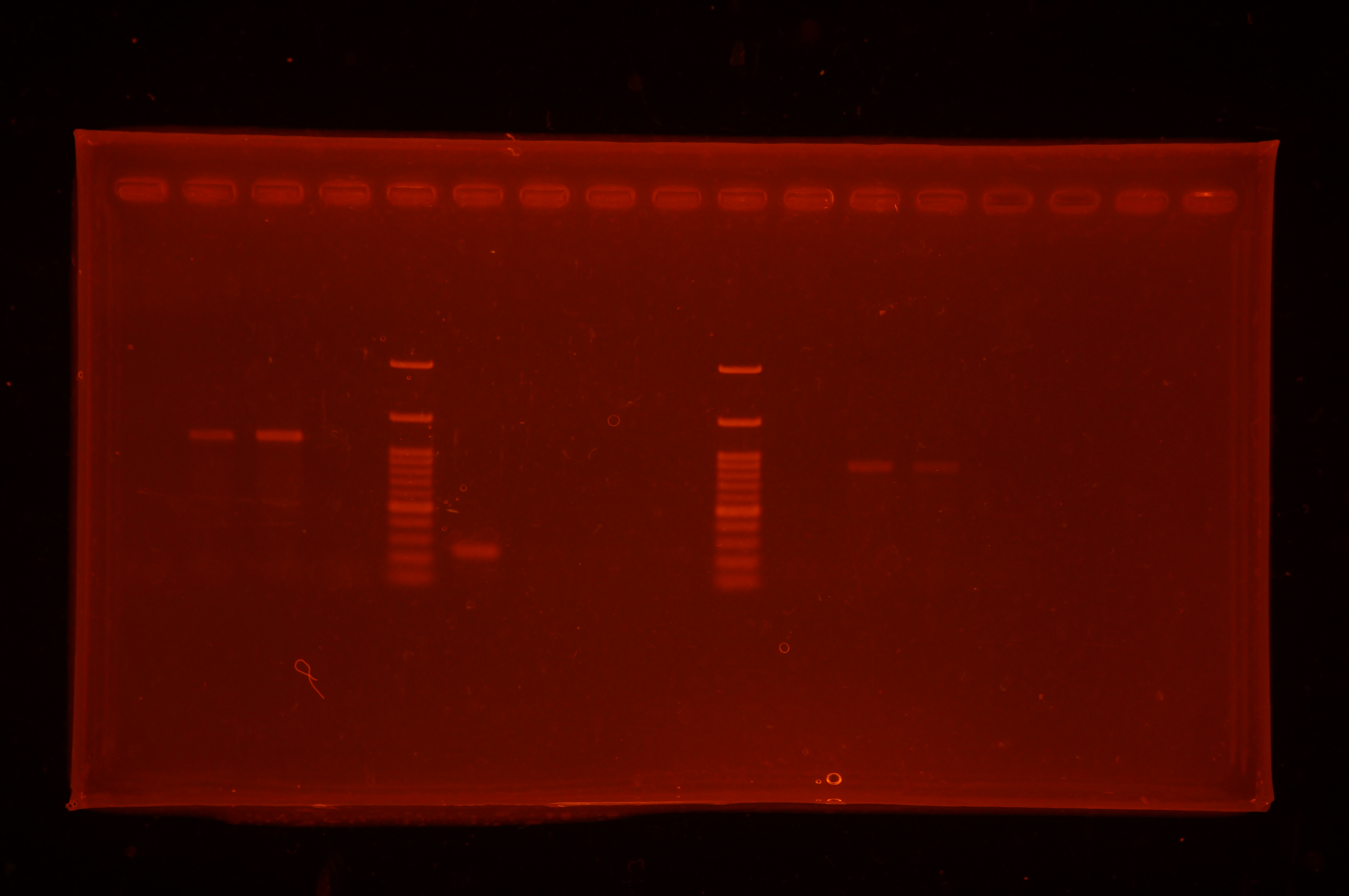

Supplement: Supplementary file 7 [file Image7.jpeg]

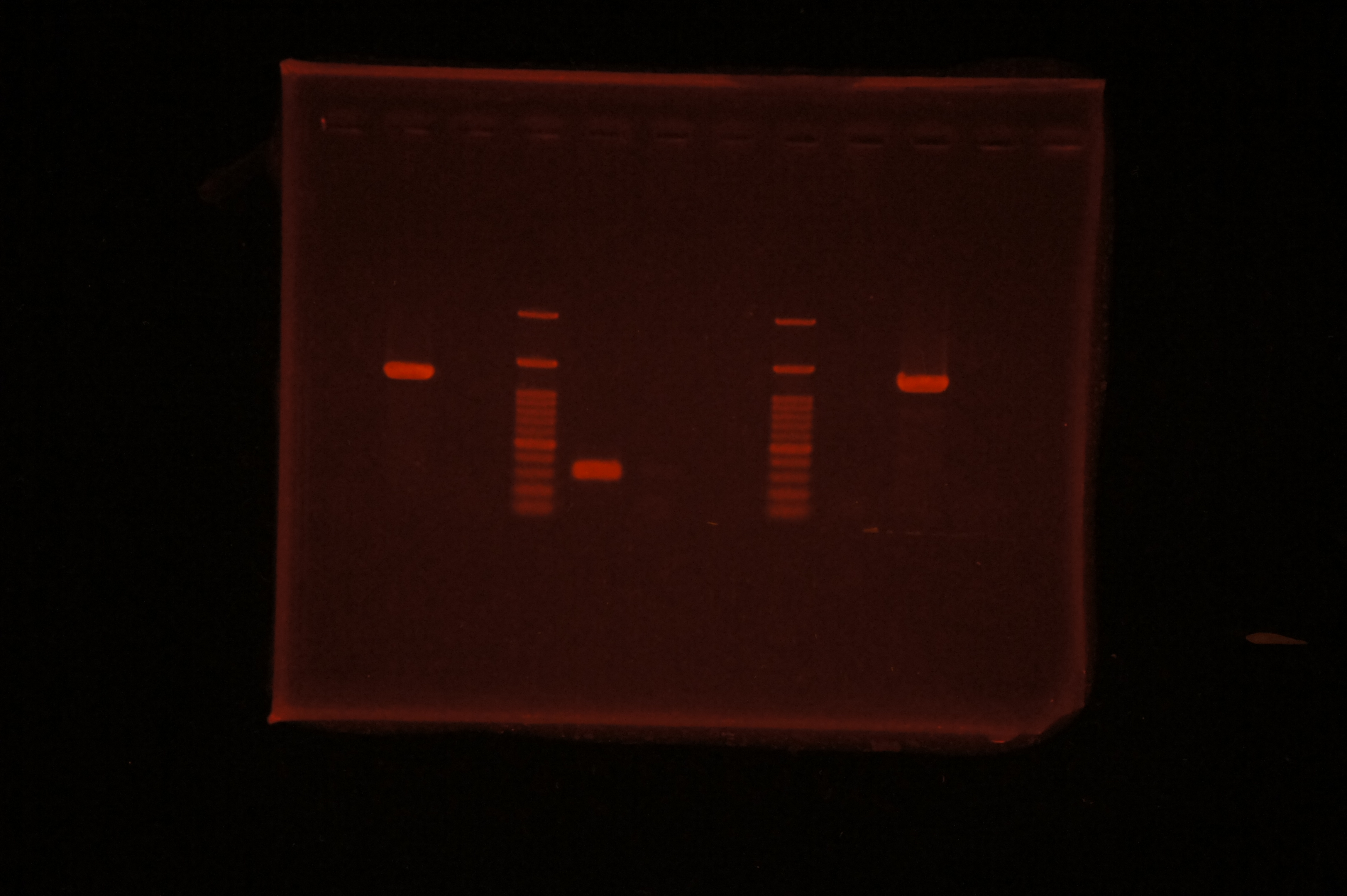

Supplement: Supplementary file 8 [file Image8.jpeg]

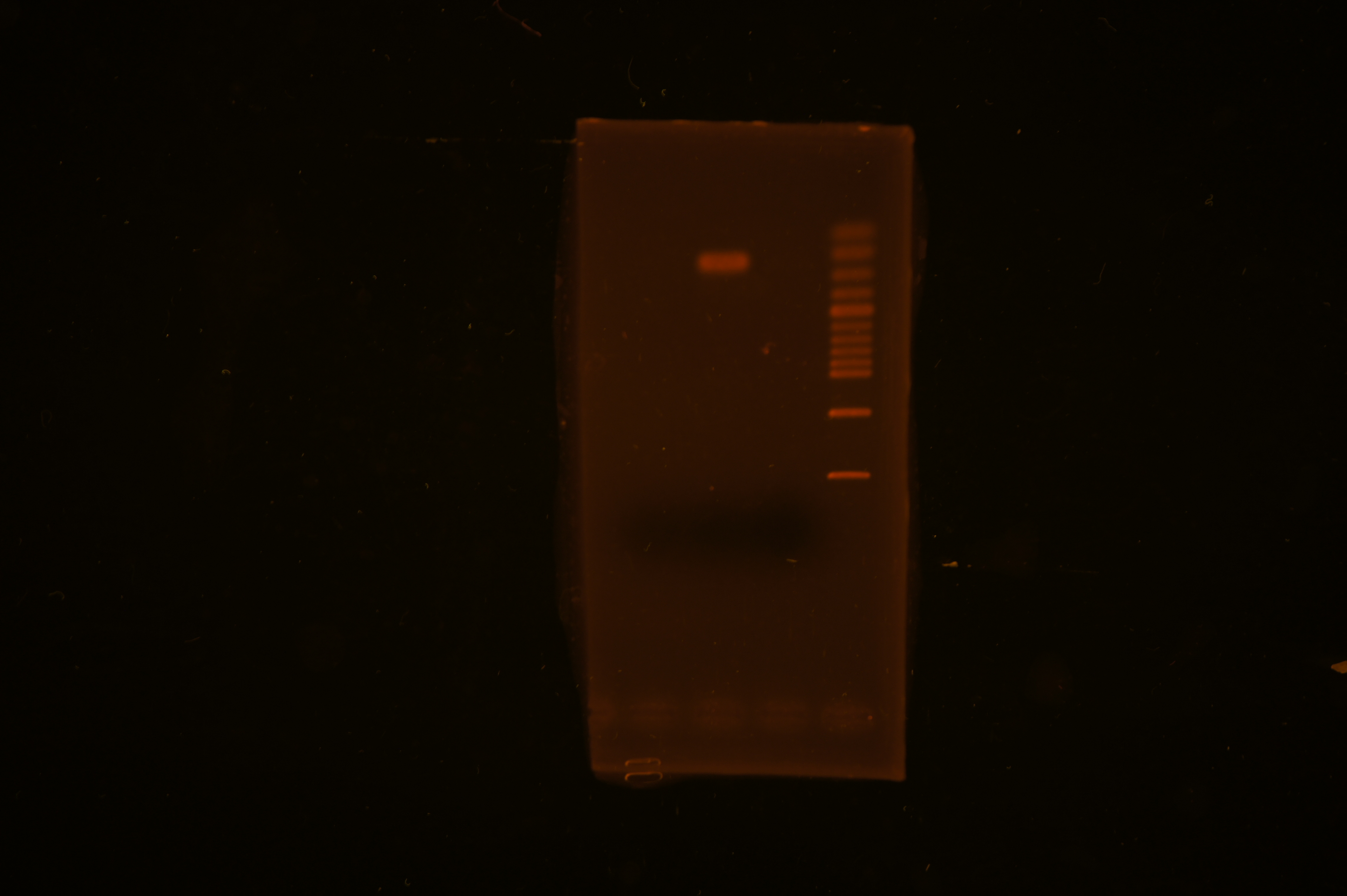

Supplement: Supplementary file 9 [file Image9.jpeg]

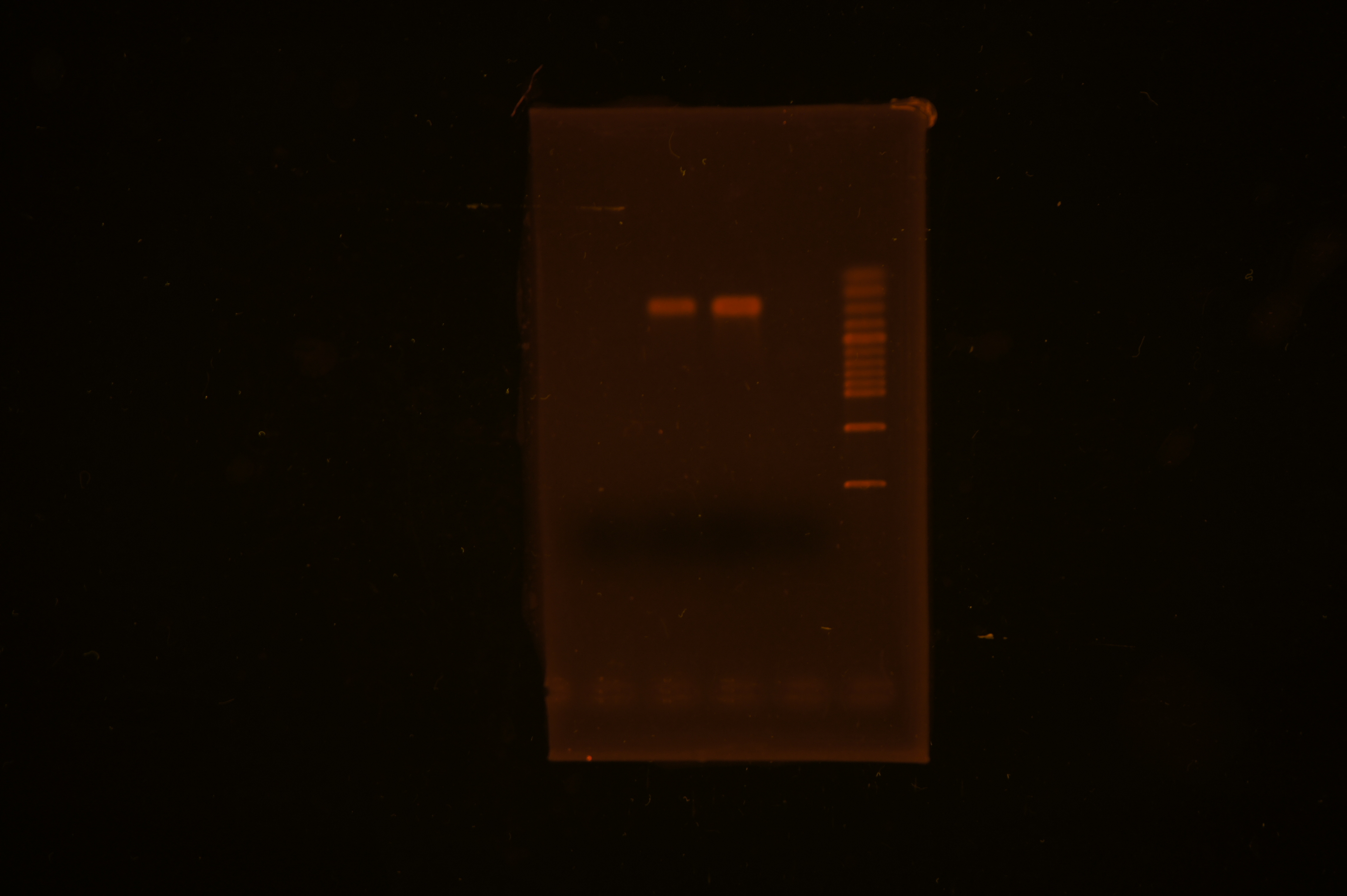

Supplement: Supplementary file 10 [file Image10.jpeg]

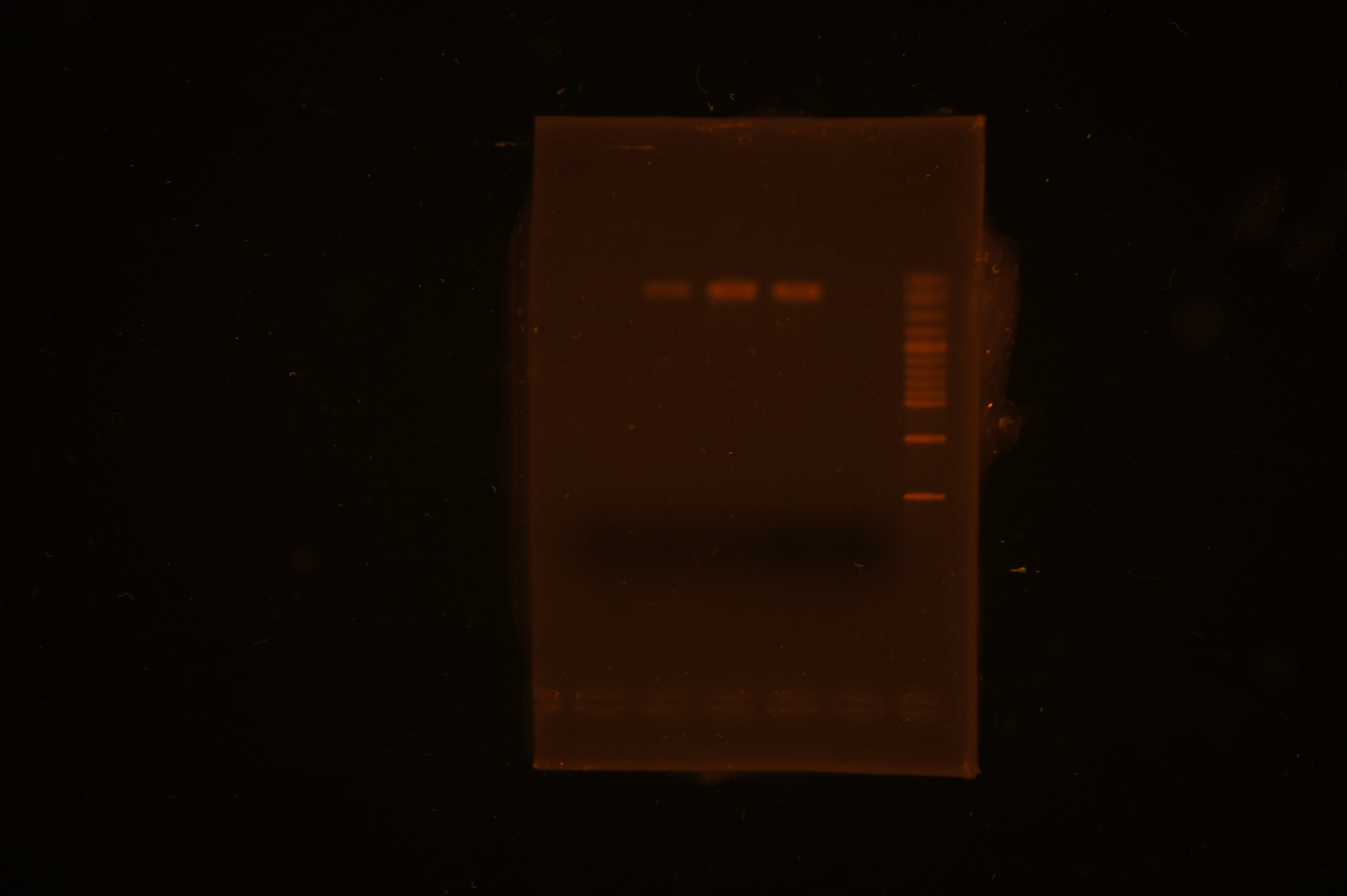

Supplement: Supplementary file 11 [file Image11.jpeg]

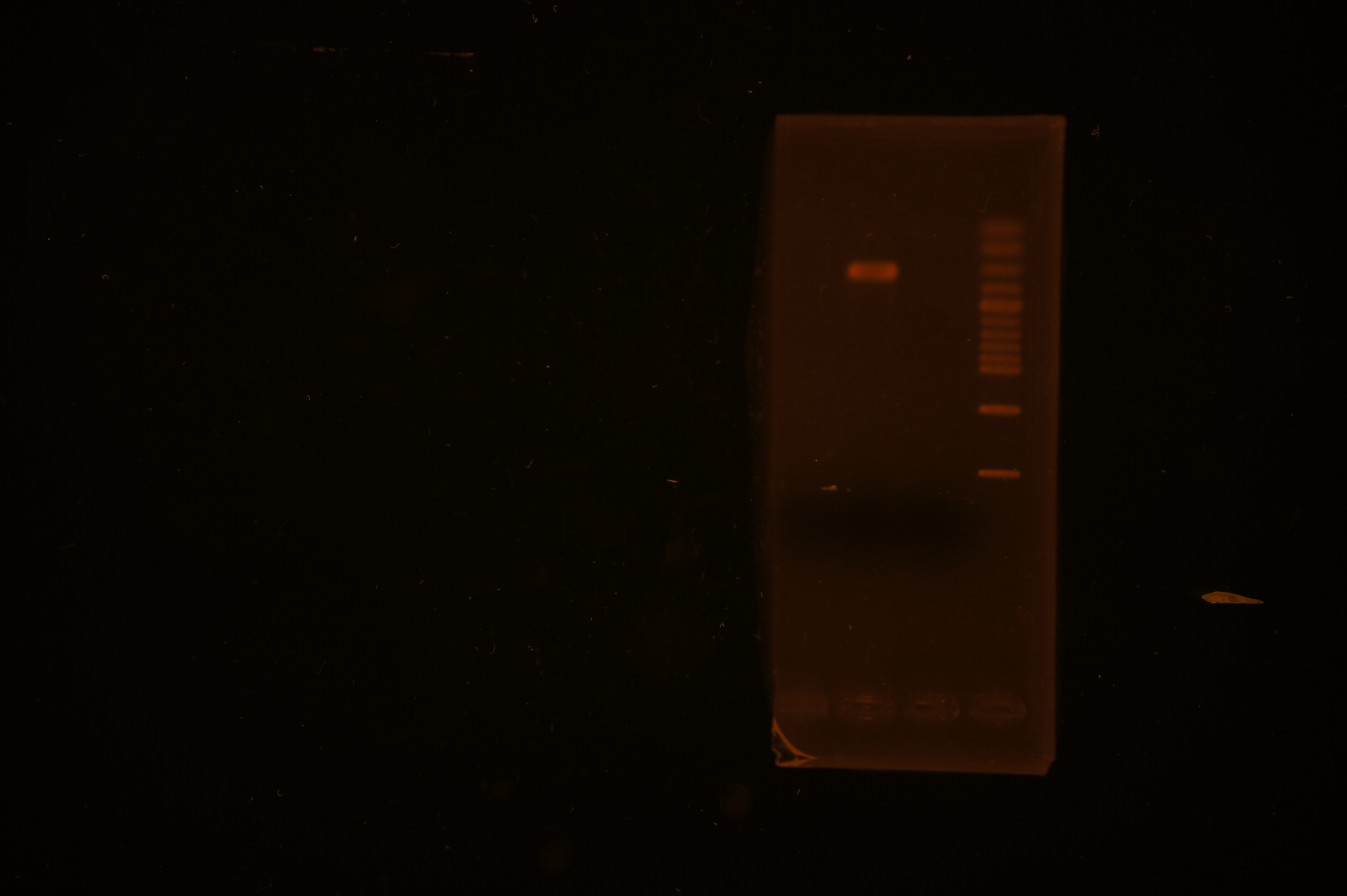

Supplement: Supplementary file 12 [file Image12.jpeg]

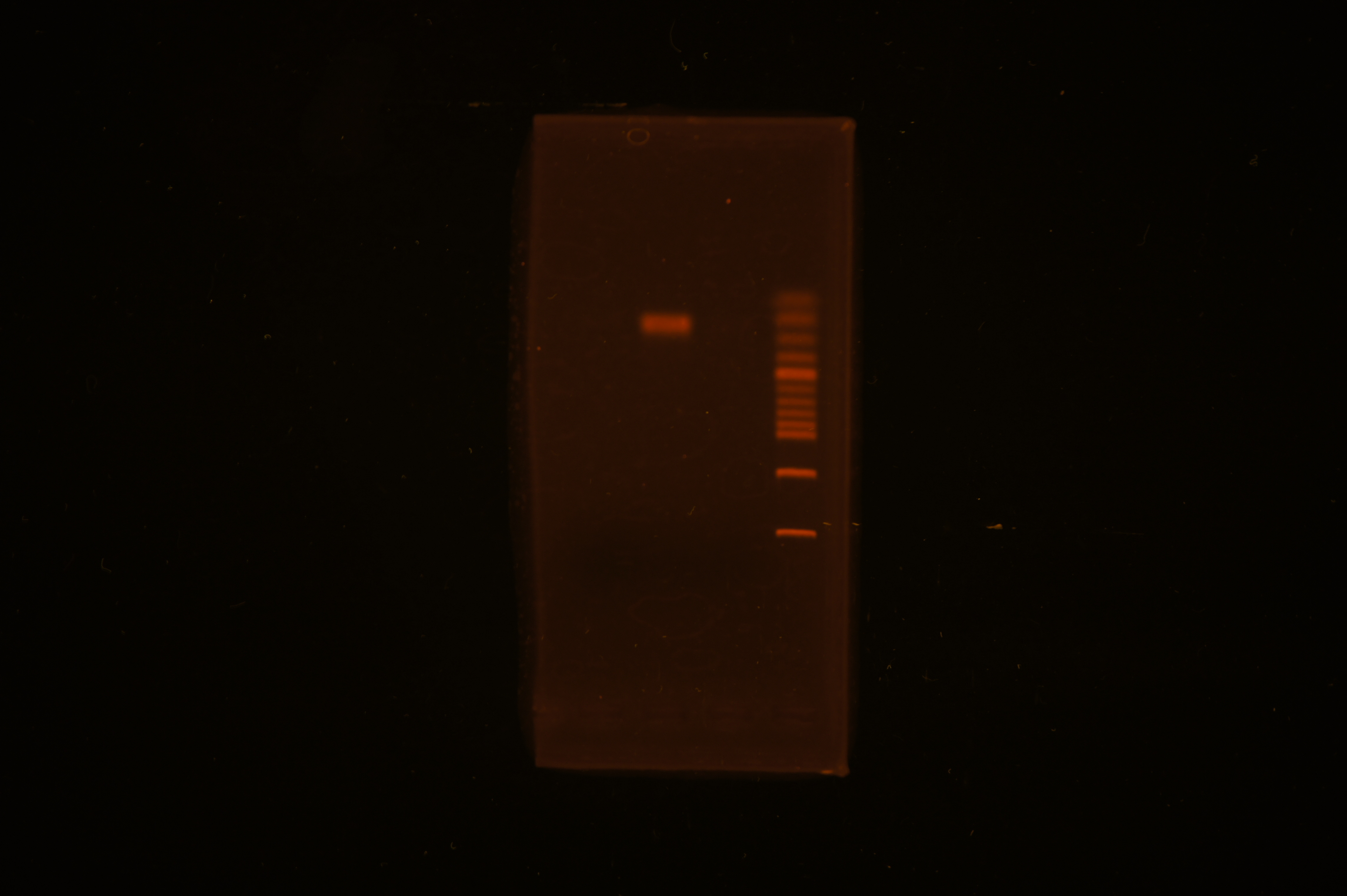

Supplement: Supplementary file 13 [file Image13.jpeg]
