# Supplementary material for: Hydrophobins in Bipolaris maydis do not contribute to colony hydrophobicity, but their heterologous expressions alter colony hydrophobicity in Aspergillus nidulans
Source: Front Fungal Biol. 2025 Nov 20;6:1604903. doi: 10.3389/ffunb.2025.1604903 (PMC12675215; doi:10.3389/ffunb.2025.1604903)
Supplement: Supplementary file 14 [file DataSheet1.pdf]

Supplementary Material

Hydrophobins in *Bipolaris maydis* do not contribute to colony hydrophobicity, but their heterologous expressions alter colony hydrophobicity in *Aspergillus nidulans*

Kenya Tsuji, Hiroshi Yoshida, Masafumi Saba, Yuki Terauchi, Moriyuki Kawauchi, Yoichi Honda, Chihiro Tanaka, Akira Yoshimi*

*** Correspondence:** Akira Yoshimi

yoshimi.akira.8c@kyoto-u.ac.jp

Supplementary Table S1. Primers used in this study

Name	Nucleotide sequence (5′–3′)
(dT)21VN	TTTTTTTTTTTTTTTTTTTTTVN
Hyp1-f1	GGCATGGCGACCAGTCTGAAAG
Hyp1-r1	CCTCAGGCATTTGAGAAGCACCTTGAAAGTAATTCAACGAGTTGGTATTG
Hyp1-f2	CTCATCATTGGAAAACGTTCTTCGCGCTACTACAGCGACATGTTG
Hyp1-r2	ACATGAACGGCTCCTTCAGATTAGC
Hyp1-f3	CGTTTCCGAACTTGTACTATGGTTAG
Hyp1-r3	TCTTGGAGACTATGTCCAGATCTTC
Hyp1-f4	CAATCCAAAGTCCGTTTTTCCCATC
Hyp1-r4	GGGTTGCAGGCGATGTTGAC
Hyp2-f1	GTCTCGCGTCCACATGCTTG
Hyp2-r1	CCTCAGGCATTTGAGAAGCACGGCTTGCGTGCTCTCTTTATAC
Hyp2-f2	CTCATCATTGGAAAACGTTCTTCGCTCGGAGTTTTCTCAGAACC
Hyp-r2	GGGATGCGCTTTGTGGGTATTATG
Hyp2-f3	CGCAGCCAAAAGATGCTTAATGTG
Hyp2-r3	GGTGTTCTTGGTCTATGATCCTTG
Hyp2-f4	TCCTGGACCTCGCTGACTTG
Hyp2-r4	GCAGGAGCAGTAGGGTTGAC
Hyp3-f1	ACACAGTAGACTACCGAGCATAC
Hyp3-r1	CCTCAGGCATTTGAGAAGCACAACTGGCAGTGCAGGTAGTG
Hyp3-f2	CTCATCATTGGAAAACGTTCTTCGAGAATACGGGCGGTGCAAG
Hyp3-r2	GCCGCGTATGAGCTGAGGTGATC
Hyp3-f3	GGCTCGGGAACAGGATCAAG
Hyp3-r3	GTACGGAGAGTAGTTTGTTGCTTG
Hyp3-f4	CTCTCCTTTGCCAGGATGTTAG
Hyp3-r4	AAGTAGGAGTCTGAACGGCTTG
Hyp4-f1	GCAATGCCACTCGGACACTTG

Supplementary Table S1 (continued)

Hyp4-r1	CCTCAGGCATTTGAGAAGCACTAGTTGGATAAGTTGGTGATGAGAC
Hyp4-f2	CTCATCATTGGAAAACGTTCTTCGAGACATGCTCTTCTGTCGATTC
Hyp4-r2	AGGGATAGCTTGGAGTTTGAGTGAAG
Hyp4-f3	AAGACCGCGCCATACTGTCATTC
Hyp4-r3	AAGCATAAGCGCGGTGGATTTATC
Hyp4-f4	GGTTTGCTGGGTGTGTCCAAC
Hyp4-r4	GCAAGAGCGCCGTGCAAATC
NPTandHPH-f1	CGAAGAACGTTTTCCAATG
NPTII-r1	TCAGAAGAACTCGTCAAGAAGGC
NPTII-chk1	CGTGATATTGCTGAAGAGCTTGG
NPTandHPH-chk2	GTTGCCTAAATGAACCATCTTGTC
NATandBAR-f1	GTGCTTCTCAAATGCCTGAGGCCAGTTTGCTCAGCGACAGAAGATGATATTGAAGG
NAT-r1	CGAAGAACGTTTTCCAATGATGAGCACTTTTTGATCGCTCAGGGGCAGGGCATGCTC
NAT-chk1	TAGCATTGATGTGTTGACCTCCAC
NAT-chk2	GGGTTTCTGGCAGCTGGACTTC
BAR-r1	CGAAGAACGTTTTCCAATGATGAGCACTTTTTGATCGACCTAAATCTCGGTGACGGGCAG
BAR-chk1	TAGCATTGATGTGTTGACCTCCAC
BAR-chk2	GGGTTTCTGGCAGCTGGACTTC
HPH-r1	GTGCTTCTCAAATGCCTGAG
HPH-chk1	TGTAGAAGTACTCGCCGATAGTGG
dewA-f1	CCTGCAGGTCGACTCTAGAGGCTAGTGGTTGTTCGCTGTATGTAAC
dewA-r1	GGCCACAGCGGATGGAATTCTGTGATGGAAGGCAGCTGATGAAG
dewA-f2	CCAGTTACTCCGTCGGTACCGTCTGGACGCTCCTAGGTATCTTG
dewA-r2	ATTCGAGCTCGGTACCCGGGCAGCGGTCAACTGCCAGTCATAC
AoargB-f1	GAATTCCATCCGCTGTGGCCGACTCA
AoargB-r1	GGTACCGACGGAGTAACTGGAAAGATACGA

Supplementary Table S1 (continued)

dewA-f3	CACTCAATCCGTGCCTACAAATGTG
dewA-r3	CGGTGCTTCAGTGTTGAGTACTTC
dewA-f4	CTGAAGGCACCACCTGCAATGTC
dewA-r4	CAGGGCAGCAAGCGACGATGTTC
AoargB-chk1	CTTCGGCAGCCTCAGAAGACTTTC
AoargB-chk2	CGATGATGTCGAGCACCAGGATTAC
pUC-chk1	GATAACAATTTCACACAGGAAACAGCTATG
pUC-chk2	CACGACGTTGTAAAACGACGGCCAG
pyrG-f1	CCTGCAGGTCGACTCTAGAGGAATTCGATACCTGTCGAA
pyrG-r1	ATTCGAGCTCGGTACCCGGGTGCTTGTCTACCAGATTAG
dewA-pyrG-f1	TGGTAGACAAGCAGATCCCCGCTAGTGGTTGTTCGCTGTATGTAAC
dewA-pyrG-r1	TGAATTCGAGCTCGGTACCCCAGCGGTCAACTGCCAGTCATAC
dewAP-HYP1-r1	AAGATAACCTTGGCGAGCATTTTGATGAGTTGAGTTGAG
HYP1-start-f1	ATGCTCGCCAAGGTTATCTTCCTCAC
HYP1-pyrG-r1	TGAATTCGAGCTCGGTACCCCCTGCTGGGTCATGCTATAATTCTG
PdewA-r1	TTTGATGAGTTGAGTTGAG
HYP2-PdewA-f1	ACTCAACTCAACTCATCAAAATGCAGTTCTCCATCACCACCATC
HYP2-pryG-r1	TGAATTCGAGCTCGGTACCCCGAGCCTCGCACCAAAGACCTTAC
HYP3-PdewA-f1	ACTCAACTCAACTCATCAAAATGCGTTACACTATCCTCGCCTTC
HYP3-pryG-r1	TGAATTCGAGCTCGGTACCCCTGCCACCAGCTCCCTCTTCAAG
HYP4-PdewA-f1	ACTCAACTCAACTCATCAAAATGCGTTTCATCCTCATTACCCTGAC
HYP4-pryG-r1	TGAATTCGAGCTCGGTACCCGTGCGACCACTACTACACCTTCATG
pyrG-chk1	GGAGCGCTTCTGTATTTCTTTGCATG

Underlines show the overlapping sequences for PCR fusion or HiFi DNA assembly

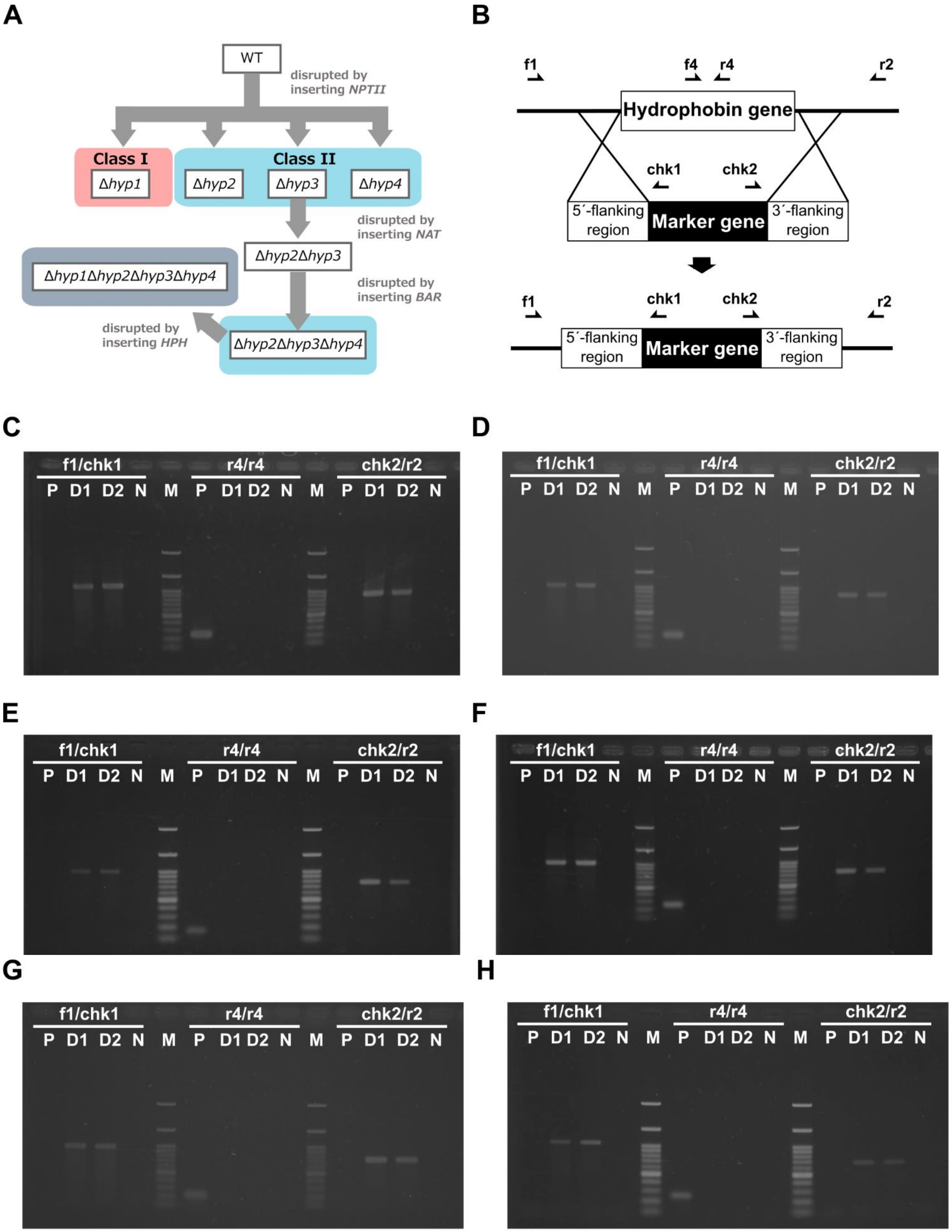
A
WT
disrupted by inserting NPTII
Class I
Δhyp1
Class II
Δhyp2
Δhyp3
Δhyp4
disrupted by inserting NAT
Δhyp2Δhyp3
disrupted by inserting BAR
Δhyp1Δhyp2Δhyp3Δhyp4
disrupted by inserting HPH
Δhyp2Δhyp3Δhyp4
B
f1
f4
r4
r2
Hydrophobin gene
chk1
chk2
5´-flanking region
Marker gene
3´-flanking region
f1
chk1
chk2
r2
5´-flanking region
Marker gene
3´-flanking region
C
f1/chk1
r4/r4
chk2/r2
P D1 D2 N M P D1 D2 N M P D1 D2 N
D
f1/chk1
r4/r4
chk2/r2
P D1 D2 N M P D1 D2 N M P D1 D2 N
E
f1/chk1
r4/r4
chk2/r2
P D1 D2 N M P D1 D2 N M P D1 D2 N
F
f1/chk1
r4/r4
chk2/r2
P D1 D2 N M P D1 D2 N M P D1 D2 N
G
f1/chk1
r4/r4
chk2/r2
P D1 D2 N M P D1 D2 N M P D1 D2 N
H
f1/chk1
r4/r4
chk2/r2
P D1 D2 N M P D1 D2 N M P D1 D2 N

I

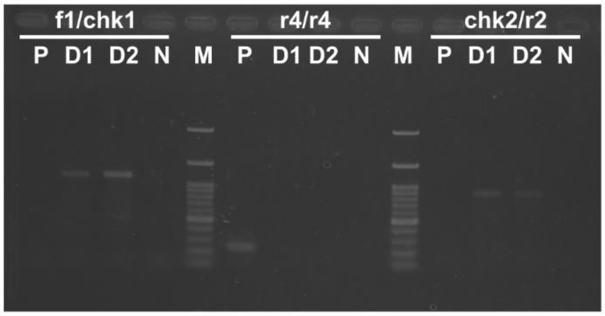

Supplementary Figure S1. Disruption of hydrophobin genes in *Bipolaris maydis*. (**A**) A strategy to generate disruptants. (**B**) A scheme of the disruption of hydrophobin genes and location of primers. (**C**–**I**) PCR to confirm disruptions with 3 sets of primers indicated at the top of each gel. The expected fragment lengths are: (**C**) Δ*hyp1*. 1138 bp, 241 bp, and 893 bp; (**D**) Δ*hyp2* 1023 bp, 221 bp, and 851 bp; (**E**) Δ*hyp3* 1020 bp, 177 bp, and 777 bp; (**F**) Δ*hyp4* 1028 bp, 258 bp, and 757 bp; (**G**) Δ*hyp2*Δ*hyp3* 975 bp, 221 bp, and 732 bp; (**H**) Δ*hyp2*Δ*hyp3*Δ*hyp4* 1068 bp, 258 bp, and 688 bp; (**I**) Δ*hyp1*Δ*hyp2*Δ*hyp3*Δ*hyp4* 1163 bp, 241 bp, and 983 bp. M, 100 bp DNA ladder marker; P, parental strain; D1 and D2, disruptants; N, negative control (TE buffer).

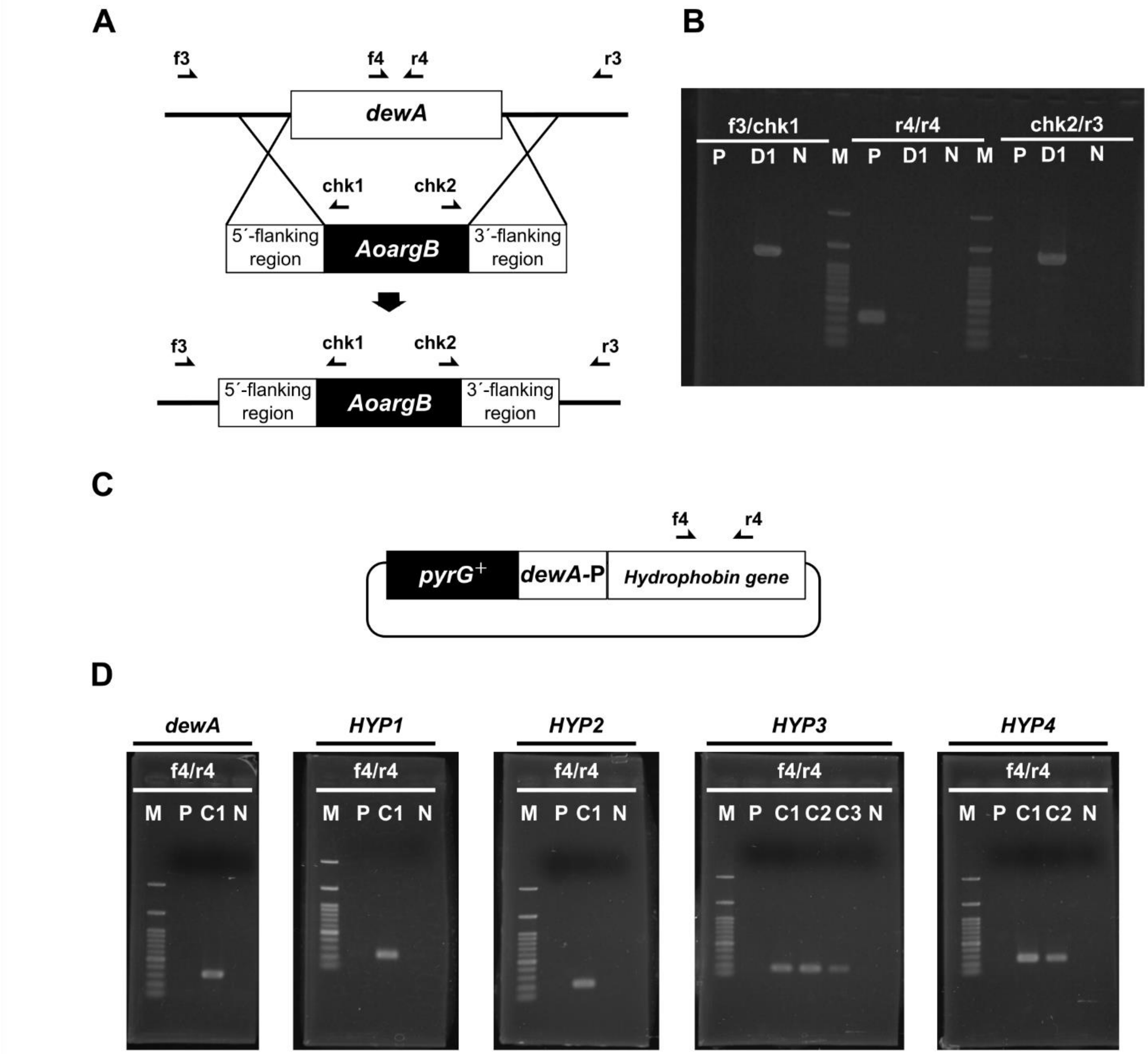

Supplementary Figure S2. Disruption of the *dewA* gene in *A. nidulans* and complementation with *dewA* or *B. maydis* hydrophobin *genes*. (**A and C**) Schemes of **(A)** disruption of *dewA* hydrophobin gene; **(C)** the plasmid for the inserion of *dewA* gene or *B. maydis* hydrophobin genes under the control of the *dewA* promoter. Locations of primers are shown in each scheme. (**B and D**) PCR to confirm the expected structures with sets of primers indicated at the top of each gel. The expected fragment lengths are: 1250 bp, 325 bp, 1228 bp in B; 325 bp, 241 bp, 221 bp, 177 bp, 258 bp in D. P, parental strain; D1, disruptant 1; C1, C2 and C3, complemented strains; N, negative control (TE buffer). M, 100 bp DNA ladder marker.

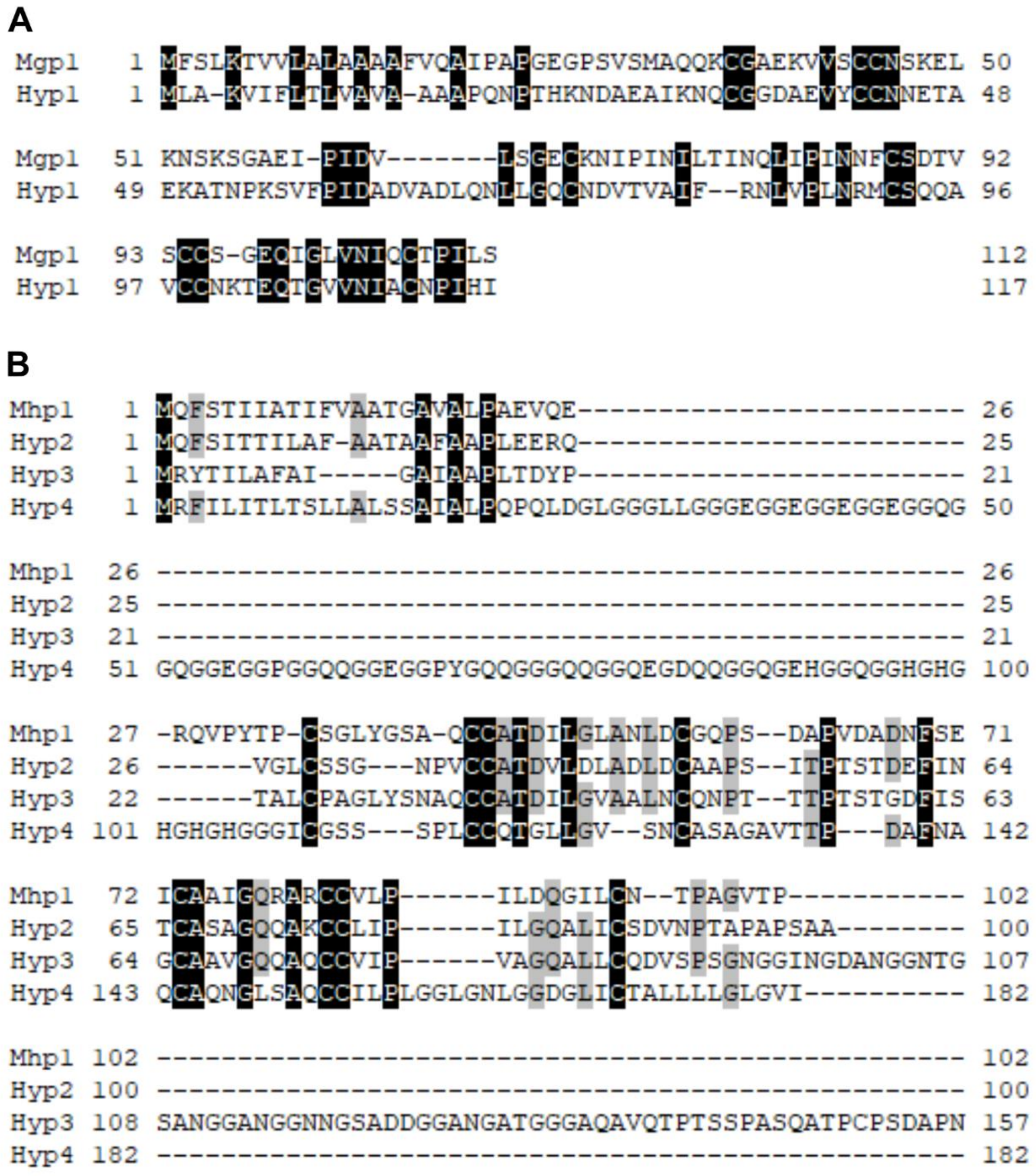

Supplementary Figure S3. Comparison of hydrophobins in *Pyricularia oryzae* and *B. maydis*. (**A**) Class I. (**B**) Class II. Unique long sequences are present at the C-terminus in Hyp3 and at the N-terminus in Hyp4. Sequences of Mpg1 (ADD84604) and Mhp1 (AAD18059) of *P. oryzae* were obtained from the NCBI database.

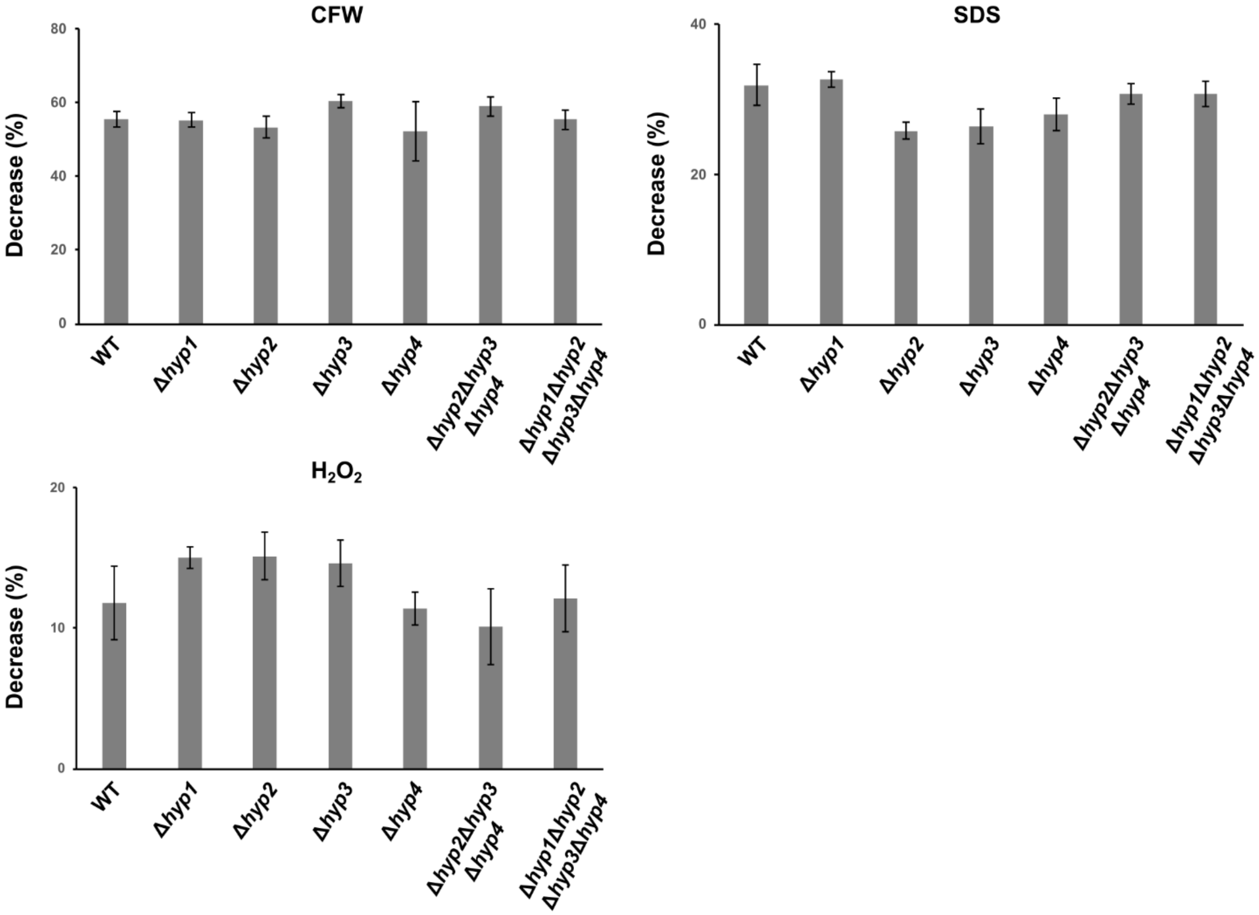

Supplementary Figure S4. Decrease in growth in each disruptant under stress conditions. The bar graphs show the decrease in colony diameter relative to the untreated control in Figure 4. Error bars, standard error (n = 3). Under each stress condition, the rate of colony diameter decrease did not differ significantly ($P \geq 0.05$) among strains in Tukey's test.

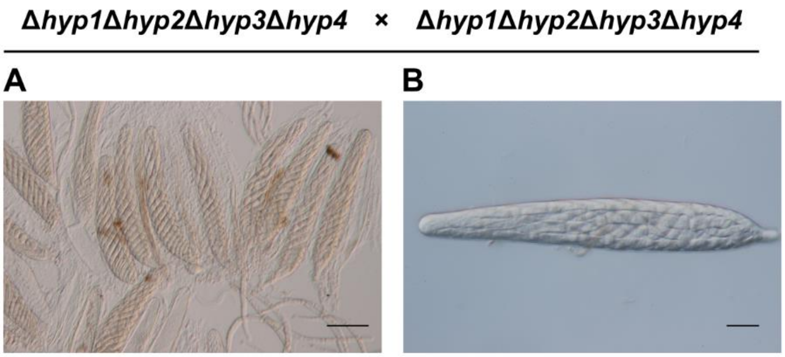

Supplementary Figure S5. Sexual reproduction of Δ*hyp1*Δ*hyp2*Δ*hyp3*Δ*hyp4*. (**A**) Asci. Bar: 50 µm. (**B**) Ascus with ascospores. Bar: 20 µm. Pseudothecia were harvested and dissected to observe the asci and ascospores at 30 days after the cross.

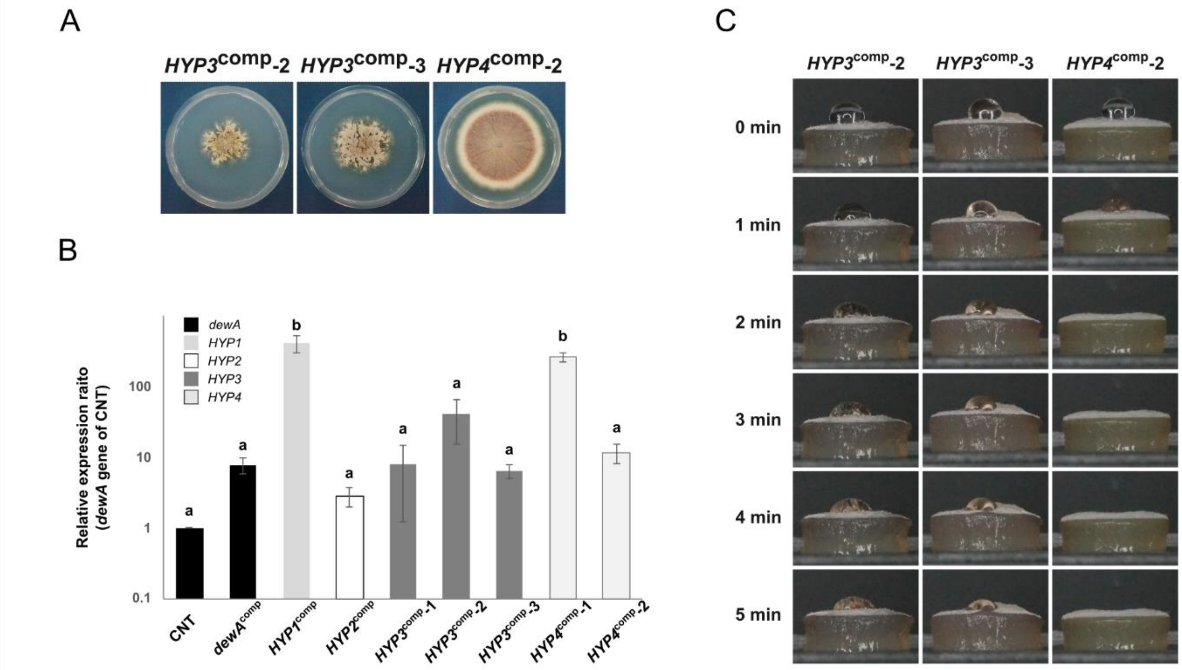

Supplementary Figure S6. Phenotypic assay of complemented strain in *A. nidulans*. (**A**) Colony growth. Each strain was incubated on CD medium for 1 week. (**B**) Transcript levels of *B. maydis* hydrophobin genes in *A. nidulans*. Mycelia from each strain were cultured for 24 hours in CD liquid. RNA was extracted and used for qRT-PCR. The dewA expression level was normalized to 1.0. Error bars, standard error (n = 3). Different letters within each gene indicate significant differences (Dunnett's test, $P < 0.05$). (**C**) Hydrophobicity of *A. nidulans* $HYP3^{comp}$ and $HYP4^{comp}$ strains. A 10-µL droplet of solution containing 0.2% sodium dodecyl sulfate and 50 mM ethylenediaminetetraacetic acid was placed on the mycelial disk of each strain and observed at the indicated time points.
